# Parallel Reaction Monitoring reveals structure-specific ceramide alterations in the zebrafish

**DOI:** 10.1038/s41598-019-56466-z

**Published:** 2019-12-27

**Authors:** Tejia Zhang, Sunia A. Trauger, Charles Vidoudez, Kim P. Doane, Brock R. Pluimer, Randall T. Peterson

**Affiliations:** 1 Department of Pharmacology and Toxicology, College of Pharmacy, University of Utah, Salt Lake City, Utah USA; 2 Small Molecule Mass Spectrometry, Harvard University, Cambridge, Massachusetts USA

**Keywords:** Lipids, Experimental organisms

## Abstract

Extensive characterisations of the zebrafish genome and proteome have established a foundation for the use of the zebrafish as a model organism; however, characterisation of the zebrafish lipidome has not been as comprehensive. In an effort to expand current knowledge of the zebrafish sphingolipidome, a Parallel Reaction Monitoring (PRM)-based liquid chromatography–mass spectrometry (LC–MS) method was developed to comprehensively quantify zebrafish ceramides. Comparison between zebrafish and a human cell line demonstrated remarkable overlap in ceramide composition, but also revealed a surprising lack of most sphingadiene-containing ceramides in the zebrafish. PRM analysis of zebrafish embryogenesis identified developmental stage-specific ceramide changes based on long chain base (LCB) length. A CRISPR-Cas9-generated zebrafish model of Farber disease exhibited reduced size, early mortality, and severe ceramide accumulation where the amplitude of ceramide change depended on both acyl chain and LCB lengths. Our method adds an additional level of detail to current understanding of the zebrafish lipidome, and could aid in the elucidation of structure-function associations in the context of lipid-related diseases.

## Introduction

The zebrafish is a versatile model organism that has shown significant promise in the study of metabolism. Zebrafish and mammals share considerable overlap in major organs and in key nodes of metabolic control such as insulin signalling and appetite regulation^1–3^. Transgenic zebrafish with tissue-specific fluorescence have been implemented in studies of pancreatic β-cell regeneration^4^, dietary cholesterol trafficking^5^, gluconeogenesis^6^, and iron metabolism^7^, while recent advances in targeted mutagenesis have contributed to the rise in zebrafish models of metabolic disorders^8–11^.

Complementing the aforementioned efforts, multiple studies have focused on defining the zebrafish metabolome in both its wild-type state^12,13^ and in the contexts of development^14–16^, regeneration^17^, and toxin response^18–20^. Ong *et al*. adopted a multi-analytical approach toward metabolomic analyses of zebrafish livers; the combination of nuclear magnetic resonance, gas chromatography–mass spectrometry (GC–MS) and liquid chromatography–mass spectrometry (LC–MS) revealed differences in amino acid and unsaturated fatty acid profiles between male and female zebrafish^21^. GC–MS and LC–MS were used by Huang *et al*. to define developmental stage-specific metabolic transitions during zebrafish embryogenesis^14^. Using a combined workflow of LC–MS/MS and HPLC with fluorescence/charged aerosol detectors, Quinlivan *et al*. obtained detailed profiles of the larval zebrafish lipidome in its wild-type state and under conditions of feeding with fluorescently labelled lipids^13^. Fluorescent lipid metabolism is in turn influenced by dietary manipulations, as the incorporation of fluorescent fatty acids into cholesterol esters is reduced in instances of decreased dietary cholesterol availability^13^. An added advantage of the zebrafish system during early development is the ease of dissociating the yolk sac from the embryo body, thus allowing study of metabolism within each of these compartments^16^. Using a targeted LC–MS approach, Fraher *et al*. analysed the zebrafish yolk sac and embryo body during the first five days of development, identifying temporal and compartment-specific changes in 365 lipids belonging to 24 lipid classes and subclasses^16^. The authors’ findings, coupled with metabolic tracing *via* delivery of fluorescent fatty acid analogues into the yolk sac, demonstrate active lipid metabolism within the yolk sac and lipid transport into the embryo body^16^.

Expansion in knowledge of the zebrafish metabolome has been largely aided by advances in analytical technology. Metabolomic studies traditionally separate into untargeted and targeted approaches^22,23^. Untargeted methods frequently employ high-resolution mass spectrometers (HRMS) that detect all ions within a predefined *m/z* range, enabling identification of both known and unanticipated metabolites^22^. Targeted methods such as Multiple Reaction Monitoring (MRM), typically conducted on a triple quadrupole mass spectrometer, detect pre-specified metabolites of interest^23^. By taking advantage of metabolite-specific fragmentations, MRM detects pre-specified fragment ions from pre-specified precursors with high sensitivity, but limited mass accuracy relative to untargeted methods^23^. In recent years, integration of targeted and untargeted instrument components has led to additional approaches such as Parallel Reaction Monitoring (PRM)^22–28^.

PRM is a targeted quantification method associated with high-resolution hybrid mass spectrometers such as the quadrupole-orbitrap and quadrupole-time of flight (QqTOF)^22–28^. The combination of quadrupole mass filter and HRMS allows metabolites from a pre-specified list to be targeted for fragmentation, yielding high-resolution MS^2^ spectra for each metabolite target^25^. The resulting MS^2^ data could be further extracted to generate ion chromatograms corresponding to different fragments originating from each targeted precursor^25^. Advantages unique to PRM include sub-parts-per-million (ppm) to ppm mass accuracy at the MS^2^ level, and the ability to quantify multiple fragments from the same precursor without the need to specify them pre-sample run^22,23,25^. Importantly, hybrid HRMS setups often provide a range of scan and multiplex options such as untargeted MS^1^, MS^1^/MS^2^ and MS^1^/PRM. The combination of high-resolution MS^1^ and PRM within the same sample run facilitates elimination of false positives by boosting the confidence of precursor and fragment ion identification. PRM platforms have been successfully designed for specific lipid classes such as cardiolipins^26^ and long chain bases^23^, as well as for large-scale -omic analyses of polar and nonpolar metabolites in mammalian cell lines^25^, human serum^28^, yeast^27^ and barley root^22^.

The adaptability of PRM toward different model organisms also supports its implementation in the zebrafish. As prior knowledge of fragment ions is not required, PRM analysis of the zebrafish lipidome could be used to identify unanticipated fragments arising from known precursors, thus allowing discovery of novel, potentially fish-specific structural isomers. Given subtle variations in lipid structure could alter lipid function^29–32^, a more thorough investigation of endogenous lipid variations could uncover unknown structure-function associations, and help define the relationship between lipid structure and disease severity in the context of metabolic disorders. As zebrafish becomes more widely used in disease modelling, targeted lipidomic analysis of disease-associated lipids in the zebrafish could also help assess the likelihood of the corresponding fish models in recapitulating human pathologies.

Several zebrafish models have been generated for lysosomal storage diseases (LSDs)^11,33,34^, severe lipid storage disorders caused by loss-of-function mutations in lysosomal enzymes^35^. A lipid class that is significantly implicated in LSDs is sphingolipids^36^. Sphingolipids comprise a major lipid class with essential functions in embryogenesis, signalling, apoptosis, immunity and membrane integrity^37–41^. Given their involvement in all major aspects of cell biology, loss-of-function mutations in almost any sphingolipid catabolic enzyme can lead to pervasive substrate accumulation, multiple-organ pathologies and early mortality^36^. Sphingolipid metabolic pathways are highly conserved across species and centre around ceramide, the core of sphingolipid metabolism from which more complex species are derived^40^. Deficiency in acid ceramidase, the enzyme responsible for ceramide breakdown, is associated with Farber disease, a fatal lysosomal storage disorder with systemic pathologies and limited treatment options^42^. Major manifestations of Farber disease include painful subcutaneous nodules that appear near joints, joint deformation, and progressive hoarseness due to laryngeal involvement; additional affected organs include the brain, lungs, liver, heart and immune system^42^. While Farber disease symptoms fall into a spectrum, the severity of most Farber disease subtypes leads to death within the first few years of life for a significant number of patients^42^.

To expand knowledge of the zebrafish sphingolipidome and facilitate future studies of lysosomal storage disease aetiology, we have developed an orbitrap-based PRM platform focusing on ceramides and generated a zebrafish model of Farber disease using CRISPR-Cas9 mutagenesis. Ceramides were chosen for the current study given their central position as the core of the sphingolipid metabolic pathway and significant involvement in multiple biological processes^40^, relatively high biological abundance^16^, and consistent fragmentation pattern that facilitates detection *via* a PRM-based approach^43–45^ ceramide parent *m/z*’s were targeted for analysis, allowing detection of 86 distinct long chain base (LCB)-acyl chain combinations across different tissue types and stages of development. In addition to expanding the current repertoire of known zebrafish ceramides, our study revealed a striking absence of most d18:2 ceramides in the zebrafish relative to mammals, and identified novel LCB- and acyl chain-specific ceramide changes in the contexts of embryogenesis and the Farber disease model.

## Results

### A parallel reaction monitoring-based approach for ceramide quantification

**Figure 1:**
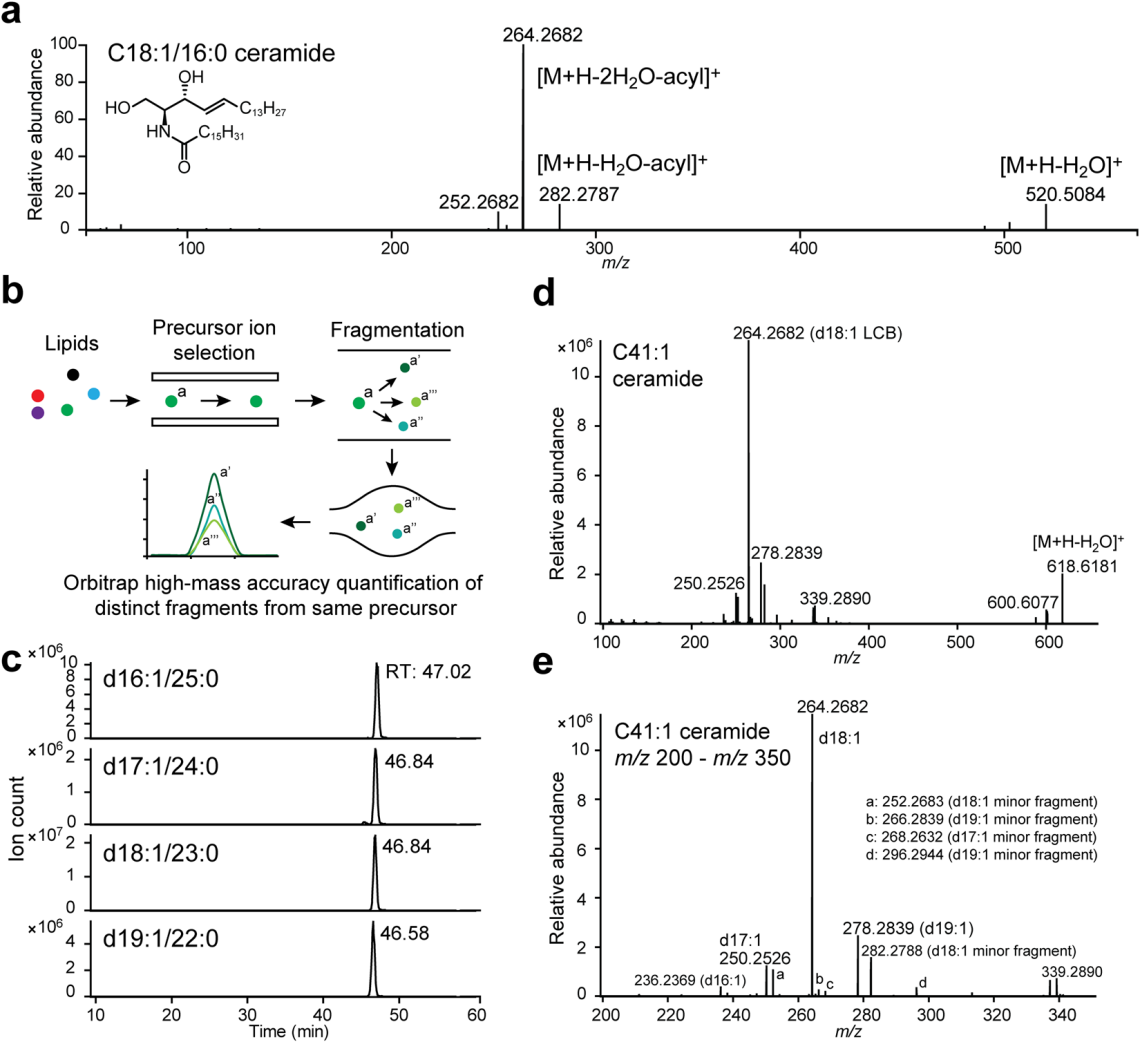
A Parallel Reaction Monitoring-based approach for ceramide quantification. (**a**) MS^2^ of d18:1/16:0 ceramide standard in positive ionisation mode. Fragment structures are based on published assignments^43,44^. (**b**) Schematic of Parallel Reaction Monitoring. (**c**) Example PRM chromatograms for C41:1 ceramide from 48 hpf zebrafish embryos, demonstrating presence of multiple LCB-acyl chain isomers with the same *m/z*. RT: retention time. (**d**) MS^2^ spectrum of C41:1 ceramide from 48 hpf zebrafish embryos, the four major LCB fragments are labelled. (**d**,**e**) *m/z* 200-*m/z* 350, the four major LCB fragments and additional LCB-derived minor fragments are labelled. Unlabelled minor fragments: *m/z* 224.2371 (d16:1 LCB), *m/z* 238.2525 (d17:1 LCB).

The positive mode fragmentation spectrum of a d18:1/16:0 ceramide standard is shown in Fig. 1a. The [M + H]^+^ parent ion undergoes sequential dehydration and loss of the acyl chain to yield three LCB-specific fragments (*m/z* 252, 264 and 282 for d18:1 ceramides)^43,44^; the initial loss-of-water ([M + H-H_2_O]^+^) fragment could also be consistently detected. This LCB-specific fragmentation pattern enables unambiguous quantification of ceramide isomers *via* targeted methods such as Parallel Reaction Monitoring (PRM). Under this approach, a pre-specified list of ceramides is targeted for fragmentation, yielding high-resolution MS^2^s for each ceramide parent ion (Fig. 1b)^24^. Extraction of LCB-specific fragments from the MS^2^ spectra of each parent ion allows quantification of LCB-acyl chain isomers sharing the same parent *m/z* (Fig. 1b)^24^. As PRM acquires the full MS^2^ for each ceramide, both known and unanticipated LCB-specific structures could be captured *via* this approach.

Based on previously analysed ceramide species^16,31,45,46^, we chose our *m/z* inclusion list to include monounsaturated and diunsaturated ceramides from C32 to C48 (Table S1). Given a peak width of ~0.5–1 minute under our current chromatography condition, the MS^2^ resolution was set at 35000 to allow ~5–10 data points per peak (for additional notes on chromatography, see Supplemental Discussion and Figs. S6–S10). To quantify ceramide isomers, *m/z*’s of the major LCB fragments ([M + H-2H_2_O-acyl]^+^) corresponding to d16:2 through d20:2, and d16:1 through d20:1 were manually extracted from the MS^2^ spectra of each targeted parent ion. Additional extractions of d14, d15, d21 and d22 LCBs did not yield significant signal. Example chromatograms and MS^2^ of C41:1 ceramide in 48 hours-post-fertilisation (hpf) zebrafish embryos are shown in Fig. 1c–e, illustrating the presence of four structural isomers with the same parent *m/z*.

### Ceramide composition in zebrafish and humans

**Figure 2:**
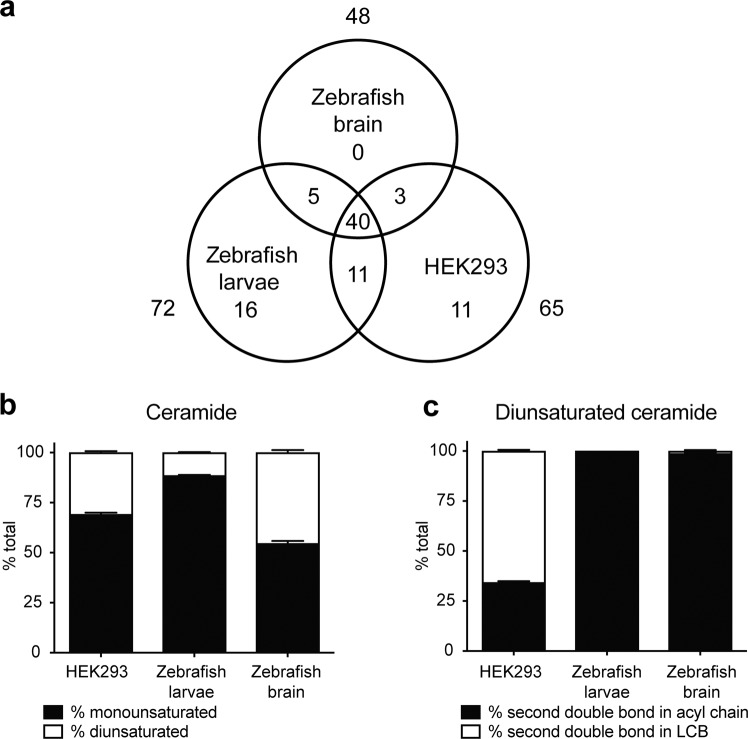
Ceramide composition of zebrafish brain, larvae and HEK239 cells. (**a**) Venn diagram illustrating total number of quantified ceramides and overlap among different sample types. (**b**) Percentage of monounsaturated and diunsaturated ceramides in HEK293 cells, zebrafish larvae and brain. (**c**) Percentage of HEK293, zebrafish larva and brain diunsaturated ceramides with the second degree of unsaturation in either the acyl chain or the LCB. SEM, n = 4 per group. Representative data from three independent experiments are shown.

To compare the ceramide profiles of zebrafish and humans, the optimised PRM protocol was applied toward adult zebrafish brain, 7 days-post-fertilisation (dpf) zebrafish larvae and a human embryonic kidney cell line (HEK293). Ceramide levels across these three sample types are summarised in Tables 1 and S2. The correlation coefficient was 0.99997 (y = 6165754.0x + 12551539.9) in the range of 100 fmol-1 nmol for the d18:1-d_7_/18:0 ceramide standard, and 0.99984 (y = 14326319.2x + 71700089.0) in the range of 100 fmol-1 nmol for the d18:1-d_7_/24:1 ceramide standard (Fig. S1). All PRM spectra were inspected and integrated manually, using the following criteria for inclusion: (1) presence of major (quantifier) LCB fragment within 5 ppm of theoretical *m/z* and within linear range based on the d18:1-d_7_/18:0 and d18:1-d_7_/24:1 standards, for all samples in data set, (2) presence of at least one additional ceramide-specific MS^2^ fragment (loss-of-water fragment and/or a minor LCB fragment) within 5 ppm of theoretical *m/z*, in most samples of data set, (3) signal-to-noise ≥10, and 4) at least three consecutive non-zero data points across integrated peak. Ceramides that fulfilled all four criteria were quantified with internal standards; species that fulfilled all criteria but fell below the linear range of the tested standards were reported as trace (tr) (Table 1). With the exception of Fig. 2a, all reported data were generated using only ceramides with non-trace values.

**Table 1:** Ceramide distribution in zebrafish brain, larvae and HEK293 cells.

Ceramide (pmol/mg protein)		Zebrafish brain	Zebrafish larvae	HEK293	Ceramide (pmol/mg protein)		Zebrafish brain	Zebrafish larvae	HEK293
C32:2	d16:1/16:1		tr		C41:2	d16:1/25:1		tr	
	d18:2/14:0			3.8		d17:2/24:0			0.4
	d18:1/14:1			tr		d17:1/24:1	15.0	6.9	4.8
C32:1	d16:1/16:0	tr	2.7	8.0		d18:2/23:0			4.8
	d18:1/14:0	tr	tr	16.7		d18:1/23:1	43.7	2.1	3.0
C33:2	d18:2/15:0			tr		d19:1/22:1	tr	tr	
C33:1	d17:1/16:0		7.2	10.8	C41:1	d16:1/25:0		0.5	tr
	d18:1/15:0		0.7	0.8		d17:1/24:0	5.6	6.7	12.1
C34:2	d16:1/18:1		tr			d18:1/23:0	50.6	17.8	21.9
	d18:2/16:0	4.8	tr	51.2		d19:1/22:0	tr	1.7	0.5
	d18:1/16:1	tr	7.4	5.0	C42:2	d16:1/26:1	tr	0.7	tr
C34:1	d16:1/18:0	tr	tr	2.5		d17:1/25:1		tr	
	d18:1/16:0	113.1	296.0	358.6		d18:2/24:0	7.8		116.8
C35:2	d17:1/18:1		2.5			d18:1/24:1	874.2	59.1	102.7
	d18:2/17:0			1.7		d20:1/22:1	tr	tr	
	d19:1/16:1		1.4		C42:1	d16:1/26:0		1.4	0.3
C35:1	d17:1/18:0	11.3	5.0	5.8		d17:1/25:0		1.1	
	d18:1/17:0	8.9	7.1	10.1		d18:1/24:0	365.8	81.9	237.6
	d19:1/16:0	tr	7.4	2.4		d19:1/23:0		0.5	
C36:2	d18:2/18:0	5.1		54.6		d20:1/22:0	tr	tr	0.4
	d18:1/18:1	23.1	5.8	7.1	C43:2	d17:1/26:1	tr	1.4	tr
C36:1	d16:1/20:0		0.7	1.9		d18:2/25:0			2.0
	d18:1/18:0	568.8	144.9	61.6		d18:1/25:1	25.6	3.6	1.9
C37:1	d17:1/20:0		2.4	0.9		d19:2/24:0			tr
	d18:1/19:0	12.0	7.7	1.0		d19:1/24:1	7.5	1.4	1.2
	d19:1/18:0	13.2	4.1	1.1	C43:1	d17:1/26:0		2.5	tr
C38:2	d16:1/22:1	3.9		1.0		d18:1/25:0	11.9	8.0	5.4
	d18:2/20:0			9.7		d19:1/24:0	tr	2.0	1.9
	d18:1/20:1	14.9	3.1			d20:1/23:0		tr	tr
C38:1	d16:1/22:0	3.8	8.3	14.8	C44:2	d16:1/28:1		tr	
	d18:1/20:0	70.2	112.6	36.3		d18:2/26:0			2.6
	d20:1/18:0	97.8	12.1	1.7		d18:1/26:1	147.5	13.3	3.6
C39:1	d16:1/23:0		5.2	5.5		d19:1/25:1		tr	
	d17:1/22:0	12.8	33.0	17.1		d20:2/24:0			0.6
	d18:1/21:0	11.0	18.2	3.3		d20:1/24:1	10.6	tr	1.1
	d19:1/20:0		4.1		C44:1	d16:1/28:0		tr	
	d20:1/19:0	tr	0.9			d17:1/27:0		tr	
C40:2	d16:1/24:1	7.6	2.5	3.3		d18:1/26:0	43.3	22.1	4.6
	d17:1/23:1		tr			d19:1/25:0		tr	
	d18:2/22:0		tr	20.5		d20:1/24:0	tr	tr	1.9
	d18:1/22:1	167.2	5.0	5.7					
	d20:1/20:1		tr						
C40:1	d16:1/24:0	4.6	3.5	14.9					
	d17:1/23:0		1.8	0.7					
	d18:1/22:0	228.8	74.4	55.1					
	d20:1/20:0	3.3	1.4						

Ceramides that fulfilled all four criteria for inclusion (see Results: Ceramide composition in zebrafish and humans) but fell below the linear range of the calibration curves (Fig. S1) were reported as trace (tr). n = 4 per group. Representative data from three independent experiments are shown.

Following data filtering based on inclusion criteria, 86 distinct LCB-acyl chain combinations were detected, 69 of which could be quantified (Tables 1 and S2). 48, 72 and 65 ceramide species were identified in zebrafish brain, larvae and HEK293 cells, respectively (Table 1 and Fig. 2a); of these species, 34, 51 and 57 were quantifiable (Table 1). 40 species were detected across all three sample types (Fig. 2a). The total ceramide content ranged from 1–3 nmol/mg protein, and the dominant LCB in all three sample types was d18:1 (Table 1), a consequence of the transfer of C16:0-CoA to L-serine *via* serine palmitoyltransferase to generate the precursor to the d18:1 LCB^40^. Monounsaturated (d16:1-d20:1 with no additional degrees of unsaturation) and diunsaturated ceramides were detected across all three sample types (Fig. 2b). Interestingly, while a significant portion of diunsaturated HEK293 ceramides contained the sphingadiene (d18:2) LCB, sphingadiene-containing ceramides were detected only in low amounts in zebrafish brain and larvae; instead, the major contributor toward the second degree of ceramide unsaturation in the zebrafish was the acyl chain (Fig. 2c). The presence of sphingadienes has been previously demonstrated in multiple model organisms and is unlikely to be specific to HEK293 cells^31,46–49^.

### Ceramide changes during zebrafish embryogenesis

**Figure 3:**
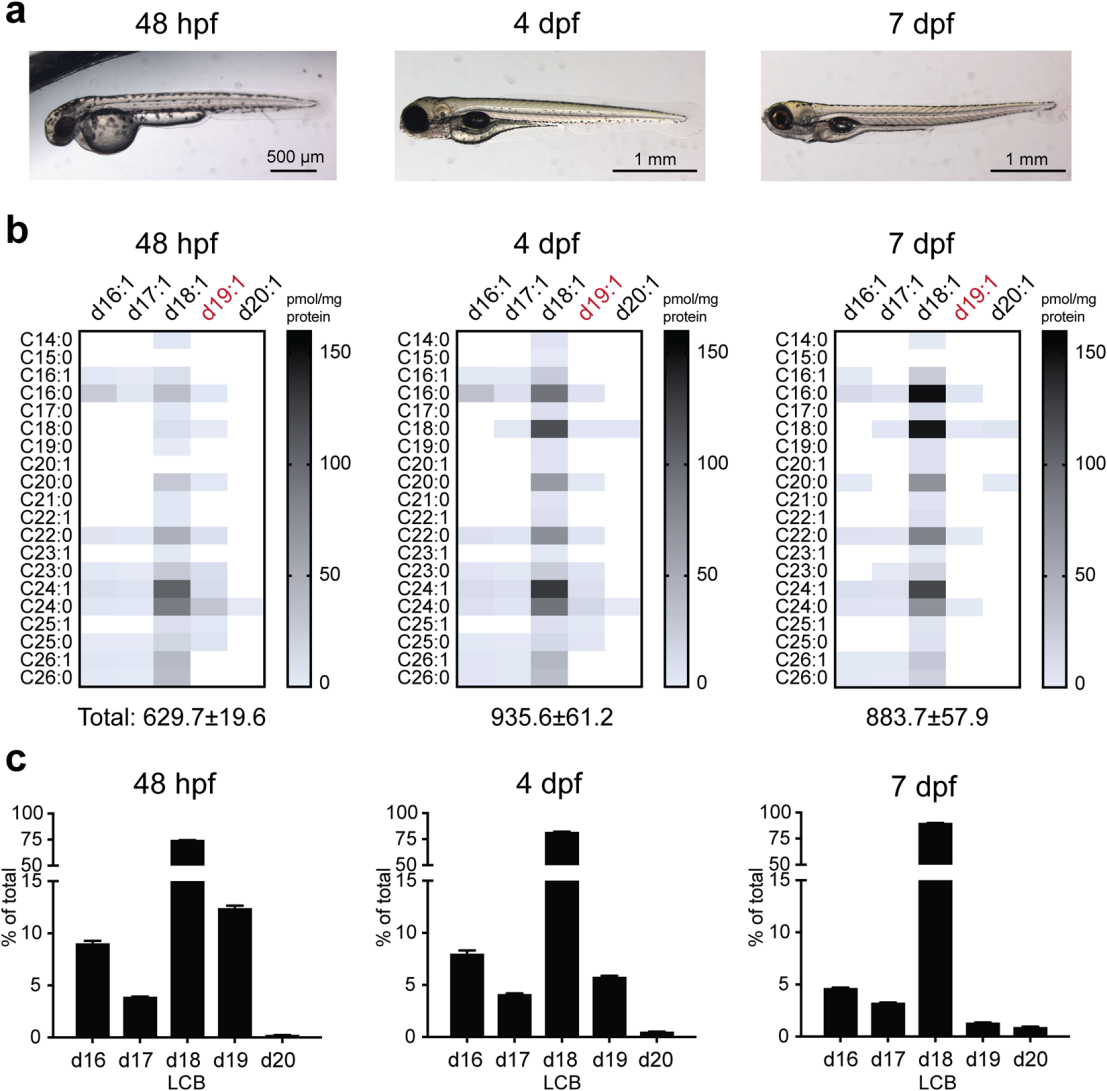
Ceramide regulation during zebrafish embryogenesis. (**a**) Zebrafish at 48 hpf, 4 dpf and 7 dpf. (**b**) Heatmap of ceramide content in 48 hpf, 4 dpf and 7 dpf larvae, demonstrating time-dependent reduction in the majority of d19:1 (highlighted in red) ceramides (see Table S3 for quantifications). (**c**) LCB distribution of all detected ceramides from 48 hpf, 4 dpf and 7 dpf larvae. n = 4 per group. Representative data from two independent experiments are shown. Additional verification of LCB distributions in 48 hpf embryos (n = 5 total) was performed *via* untargeted lipidomics (Supplemental Methods).

Having defined the zebrafish ceramide profile relative to a human cell line, we proceeded to examine ceramide regulation in the context of early zebrafish development. Following initial stages of embryogenesis, hatching occurs between ~48–72 hpf, with organogenesis continuing into the larval stage (Fig. 3a)^50^. While prey-seeking behaviour is observable by ~4–5 dpf^51^, the developing larvae also rely on the yolk sac for nutrients, which is gradually depleted and rarely visible by 7 dpf (Fig. 3a). In the absence of external food source, yolk depletion triggers the initiation of a gluconeogenic program to accommodate increasing energy demand^6^.

As zebrafish development is accompanied by significant changes in energy expenditure and therefore metabolism, we used our PRM method to capture potential development stage-specific ceramide differences. Lipids from 48 hpf embryos, 4 dpf and 7 dpf larvae were extracted and subjected to PRM analysis. The majority of the previously detected ceramides (Table 1) were present across all three timepoints, with an increase in several d18:1 species over time (Fig. 3b and Table S3). Surprisingly, a significant time-dependent reduction of d19:1 ceramides was also detected (Fig. 3b and Table S3). The fraction of d19:1 ceramides decreased from 12% at 48 hpf to 1% at 7 dpf, accompanied by a rise in d18:1 ceramides from 74% to 90% (Fig. 3c). A smaller reduction (9% at 48 hpf to 5% at 7 dpf) was also observed for d16:1 ceramides (Fig. 3c). Taken together, these data point toward LCB-specific differences in ceramide profile during zebrafish embryogenesis.

### A zebrafish model of farber disease

While we have successfully quantified ceramides in wild-type zebrafish, the ease of genetic manipulation in this model organism supports the development of ceramide-related disease models. Farber disease is a lysosomal storage disorder characterised by loss-of-function mutations in acid ceramidase (ASAH1) that lead to ceramide accumulation, multiple-organ pathologies and early mortality^42^. Morpholino knockdown of the ASAH1 orthologue in zebrafish causes loss of motor neuron branching and increased cell death in the spinal cord^52^. To gain a more thorough understanding of the metabolic landscape in the context of Farber disease, we generated a zebrafish model of Farber disease using CRISPR-Cas9 mutagenesis.

Two zebrafish orthologues (*asah1a*, *asah1b*) of human *ASAH1* were identified by database search^53^. The existence of two orthologues is a likely consequence of the additional round of whole genome duplication that occurred in teleosts relative to other vertebrates, such that 26% of zebrafish genes exist as ohnologue pairs^54^. Amino acid identity was 61% between Asah1a and ASAH1, and 62% between Asah1b and ASAH1^55,56^. To maximise mutagenesis efficiency, five CRISPR guides were designed against each of the two zebrafish ohnologues, and the mixture of ten guides was microinjected into embryos at the 1-cell stage. The guides were designed to be near residues G230 and R249 of Asah1a (NP_001006088), and G235 and R254 of Asah1b (NP_956871), as these are the conserved residues of G235 and R254 in human ASAH1 (NP_808592)^57^; G235R and R254G mutations in ASAH1 are associated with significant loss of enzyme activity and varying degrees of disease severity^58–61^.

**Figure 4:**
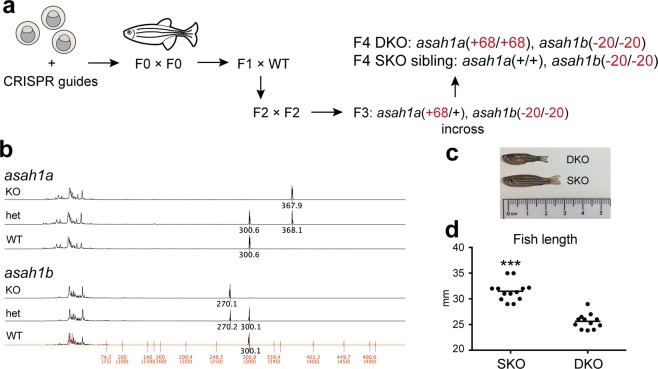
A zebrafish model of Farber disease. (**a**) Schematic of *asah1a/b*^−/−^ zebrafish generation and propagation. (**b**) DNA fragment analysis demonstrating presence of altered alleles. The 30-bp difference in the *asah1b* spectra (rather than the noted 20-bp deletion) is due to presence of different polymorphisms across the introns of the WT and KO alleles (Fig. S2b, see Fig. S3 for sequencing of full cDNA). (**c**) *asah1a/b*^−/−^ (DKO) and *asah1b*^−/−^ (SKO) zebrafish at 3.5 months. (**d**) Length of DKO and SKO zebrafish at 3.5–4 months. Zebrafish length measurements: Student t-test, SEM, combined data from n = 13 SKO and n = 12 DKO fish, fish within each data set (n = 4–5 SKO, n = 4 DKO) are age-matched. ***p < 0.001.

Initial DNA fragment analysis of ten injected embryos identified significant base pair shifts in all ten embryos, supporting a high efficiency of mutagenesis. Adult founder fish from the injected embryos were in-crossed to generate F1 fish. Following DNA fragment analysis and Sanger sequencing, F1 fish carrying mutations of interest were individually out-crossed with wild-type zebrafish to yield doubly heterozygous F2 populations (Fig. 4a). In-cross of F2 populations did not result in any surviving doubly homozygous (DKO) zebrafish, but did yield a mixture of genotypes including *asah1a*(+/−), *asah1b*(−/−), which were in-crossed to yield *asah1a*(−/−), *asah1b*(−/−) (DKO) fish and *asah1a*(+/+), *asah1b*(−/−) (SKO) siblings (Fig. 4a). The isolated mutations included a 20-bp nonsense deletion in *asah1a* and a net 68-bp insertion in *asah1b*, both of which alter the two residues involved in disease (Figs. 4b, S2 and S3). All mutations were confirmed by DNA fragment analysis (Fig. 4b), and Sanger sequencing of the genomic DNA (Fig. S2) and cDNA (Fig. S3).

Relative to SKO siblings, all DKO zebrafish exhibited significantly reduced size (Fig. 4c,d) and early mortality at ~4 months. Mutagenesis of either *asah1a* or *asah1b* alone did not lead to size or lifespan differences relative to wild-type fish, suggesting that zebrafish Asah1a and Asah1b carry overlapping functions.

### Ceramide alterations in farber disease

**Figure 5:**
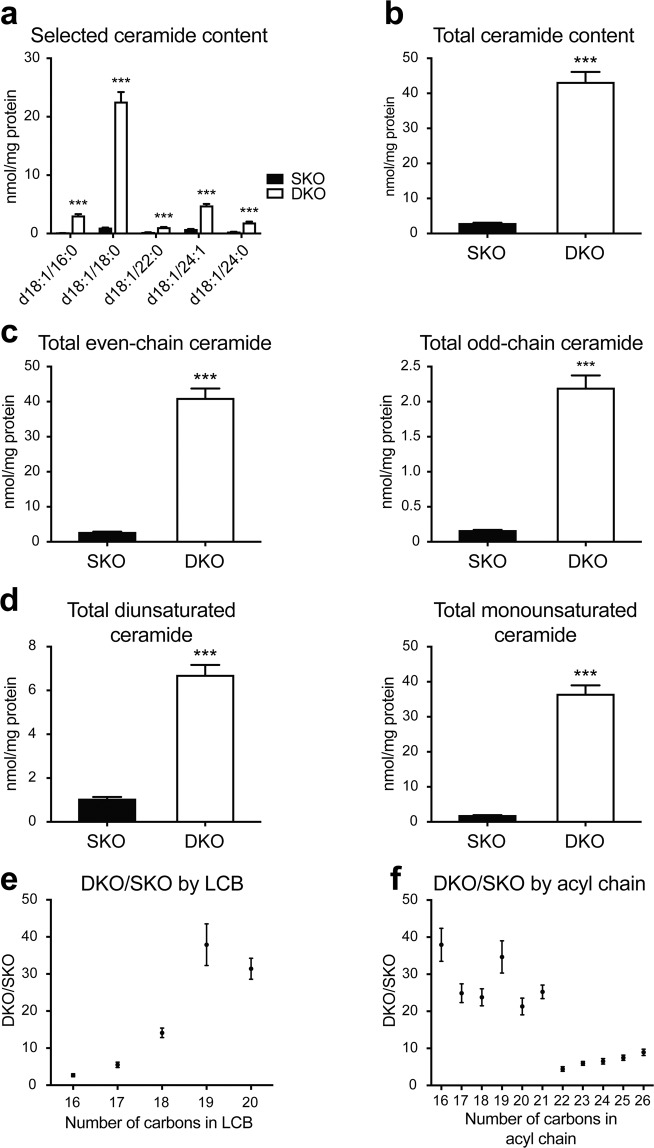
Altered ceramide distribution in Farber disease zebrafish. (**a**) Quantification for selected ceramides from 3.75-month DKO and SKO zebrafish brains (see Table S4 for quantification of additional ceramide species). (**b**) Total ceramide content in 3.75-month DKO and SKO zebrafish brains. (**c**) Total ceramide content by even and odd carbon number. Even: even number of carbons in both acyl chain and LCB; odd: odd number of carbons in acyl chain and/or LCB. (**d**) Total ceramide content by degrees of unsaturation. Monounsaturated ceramide: one double bond in LCB; diunsaturated ceramide: one additional double bond relative to monounsaturated species. (**e**) DKO/SKO fold change by LCB length. (**f**) DKO/SKO fold change by acyl chain length. Student t-test, SEM, n = 4 per group. Representative data from two independent experiments are shown ***p < 0.001.

To characterise the ceramide profile within the Farber disease model, brains were isolated from 3.5-month SKO and DKO zebrafish and subjected to PRM analysis. Significant ceramide accumulation was present for all detectable ceramides in the Farber disease model; representative species are shown in Fig. 5a (see Table S4 for additional ceramide quantifications). Total ceramide content was elevated by 15-fold in DKO zebrafish relative to SKO siblings (Fig. 5b). No change in ceramide content was detected in SKO fish relative to wild-type siblings (Fig. S4a,b), suggesting that preservation of any one of the two duplicate Asah1 enzymes is sufficient for maintaining physiological ceramide levels.

We also examined potential alterations in total monounsaturated, diunsaturated, even- and odd-chain ceramides. While total even- and odd-chain ceramides were elevated to similar degrees in DKO relative to SKO fish (Fig. 5c), total monounsaturated ceramides were increased to a larger degree (20-fold) than diunsaturated species (6-fold) (Fig. 5d). As our study is limited to brain, it is currently unknown if preferential elevation of monounsaturated ceramides in the zebrafish Farber disease model is tissue-specific.

Given our observation of LCB-dependent ceramide changes during zebrafish embryogenesis, we also examined the effects of LCB and acyl chain lengths on ceramide content in Farber disease. Interestingly, the amplitude of ceramide accumulation was LCB-dependent, as ceramides with longer LCBs were more elevated in DKO fish compared to those with shorter LCBs (Figs. 5e and S5a). In the case of acyl chain lengths, no consistent trends were observed for C14-C21 ceramides, but ceramides with C22 or longer acyl chains did not accumulate as much as C14-C21 species (Figs. 5f and S5b,c). Taken together, our data reveal that ceramide accumulation in the Farber disease zebrafish model is heavily biased toward species with longer LCBs and shorter acyl chains (Fig. S5c).

## Discussion

A PRM method was optimised for detailed ceramide isomer quantification in zebrafish and mammalian samples. 45 ceramides were targeted for PRM analysis. Quantified LCBs ranged from d16 to d20, and acyl chains from C14 to C26. Taken together, 86 distinct LCB-acyl chain combinations were detected across different sample types and developmental stages.

Total ceramide content of HEK293 cells was 1.3 ± 0.1 (±SEM) nmol/mg protein (Table 1) and within range of known values for mammalian cells (0.4–5.5 nmol/mg protein)^31,44,62–64^. Studies of zebrafish embryos, larvae and adult tissues have previously demonstrated the presence of over 30 ceramide species^16,31,65^. Total even-chain (C14-C26) ceramide content of TuAB zebrafish brain has been reported at 1.4 nmol/mg^65^, while a total brain ceramide content of 3.0 ± 0.2 nmol/mg protein was observed in our samples (Table 1); the observed differences could be attributable to potential differences in fish age and diet, as well as in the number of ceramides targeted. Our total ceramide content for 7 dpf zebrafish was 1.0 ± 0.1 nmol/mg protein (Table 1), or 145 ± 7 pmol/15 larvae. Previous measurements in 6 hpf-7 dpf zebrafish ranged from ~30 pmol/15 embryos to ~22 nmol/15 larvae^16,41,66^. These variabilities may be partially explained by differences in the ceramides targeted, larval age and strain, but could also be a consequence of sample processing. As both ionisation and extraction efficiencies depend on lipid structure, the use of different internal standards across different studies could lead to variability in quantification. Differences in chromatography and sample complexity, the latter a consequence of the extraction method, may also lead to different matrix effects around the analytes of interest and changes in signal response^67^.

Comparison of PRM data from zebrafish brain, larvae and HEK293 cells demonstrated a remarkable degree of species overlap, but also revealed some surprising differences such as the absence of the majority of d18:2 ceramides in the zebrafish. Sphingadiene-containing ceramides have been identified in multiple organisms including humans, mice, fruit flies and plants^31,46–49^; therefore, their absence in the zebrafish is unexpected and intriguing. To the best of our knowledge, this is the first demonstration of the sparsity of the sphingadiene LCB in the zebrafish model organism.

The first committed step of *de novo* ceramide biosynthesis involves the transfer of C16:0-CoA to L-serine *via* serine palmitoyltransferase (SPT) to generate 3-keto-dihydrosphingosine, which is reduced to dihydrosphingosine and then acylated by ceramide synthase to generate dihydroceramide; a 4,5-*trans* double bond is added by dihydroceramide desaturase as the last step of ceramide biosynthesis to yield mature ceramide^40^. While C16:0-CoA is the major SPT substrate, additional acyl-CoAs are also tolerated, including C16:1-CoA, which would ultimately give rise to the d18:2 ceramides that are present in mammals but mostly absent in zebrafish. Importantly, SPT catalytic activity depends on multiple subunits. In humans, SPTLC1 dimerises with either SPTLC2 or SPTLC3 to generate a fully functional enzyme complex, while two additional small activating subunits, SPTSSA and SPTSSB, enhance basal SPT activity and confer acyl-CoA specifity^68,69^. All five human SPT subunits have orthologues in the zebrafish, with amino acid identities of 61–85% (Table S5)^55^. Given different heterotrimers of SPTLCs and SPTSSs exhibit distinct acyl-CoA preferences, structural differences between human and zebrafish SPTs, as well as potential differences in subunit combinations, may lead to intolerance for the C16:1-CoA substrate and therefore the near absence of the d18:2 LCB in zebrafish. While the above hypothesis assumes origin of d18:2 ceramides from the C16:1-CoA substrate, there also exists the possibility of undiscovered sphingolipid or fatty acid desaturases capable of forming double bonds along different positions of the LCB^47^. Expansion of our current method toward the rest of the sphingolipidome could also help address the question of whether lack of d18:2 ceramides in the zebrafish is due to increased incorporation into additional sphingolipids.

While sphingadienes with 4,5-*trans* and 14,15-*cis* double bonds (Δ^4,14^) have been identified in mammals^49,70^, Δ^4,6^-sphingadienes are found in fruit fly^47^. Lipidomic analysis of the sphingosine-1-phosphate lyase-deficient *Drosophila* line *Sply* revealed preferential upregulation of Δ^4,6^-sphingadienes in the thorax, where the degenerating flight muscles associated with Sply knockdown are located^47,71^. More than 90% of all sphingadiene-containing ceramides identified in human fibroblasts were found in the detergent-soluble fraction, suggesting that these lipids may function differently from the known role of ceramides as components of detergent-resistant lipid rafts^31^. Importantly, sphingadienes promoted apoptosis and autophagy of colon cancer cells and neuroblastoma *via* modulation of Akt signaling^72,73^. Given the biological relevance of sphingadienes in mammals and fruit fly, the near absence of these species in zebrafish implies the existence of alternative lipid processing and signalling pathways that are worth exploring if the zebrafish were to be further implemented toward the study of human metabolic disorders.

Our PRM analysis of zebrafish embryogenesis identified time-dependent increase of d18:1 ceramides, and reduction of d19:1 and d16:1 species. Sphingolipids are essential for all major aspects of cell function, and disrupted sphingolipid metabolism is associated with defects in organogenesis^74,75^. Zebrafish ceramides fluctuate over the first few days of development, rising in the embryo body while decreasing in the yolk^16^; however, LCB-specific ceramide changes in the context of embryogenesis are not well understood. Our PRM analysis of zebrafish embryogenesis identified time-dependent increase of d18:1 ceramides, and reduction of d19:1 and d16:1 species. Given ceramides with different acyl chains may exert opposing functions in apoptosis^76^, our findings suggest that subtle variations in LCB length may also lead to different functions during development.

It is worth noting that as our sample isolation does not separate the yolk from the embryo body, it remains unclear if LCB length confers distinct cellular localisations; loss of d16:1 and d19:1 ceramides could thus be a direct readout for yolk depletion. Prevalence of the d18:1 LCB in mammals and zebrafish suggests that the 18-carbon length may be preferred in the context of cellular function, and more d18:1 ceramides may be required as development progresses. As the purpose of the egg yolk is predominantly fuel source, initial demands for specific LCB lengths may be less stringent in an effort to maximise yolk lipid content. As development continues, catabolism of yolk lipids leads to the biosynthesis of new d18:1 ceramides and additional, more structure-specific lipids to better suit the needs of the growing organism.

Given the necessity of ceramides for cellular function, mutations in enzymes of ceramide metabolism often result in fatal health outcomes. Reduced size and early mortality were observed in our zebrafish model of acid ceramidase deficiency (Farber disease). Size difference between *asah1a/b*^−/−^ (DKO) and SKO populations is progressive and not apparent prior to three months of age, suggesting that this phenotype may be a consequence of progressive ceramide accumulation that significantly compromises health upon reaching a threshold. Given the frequent occurrence of progressive joint deformations and contractures in Farber disease^42^, reduced size in the DKO zebrafish may be indicative of impaired skeletal development; small animal imaging techniques such as microCT have been successfully optimised for the zebrafish and could prove helpful toward addressing this question^77^. It is also worth noting that unlike other lysosomal storage disease models^11,33^, the majority of DKO zebrafish do not develop cachexia or progressive loss of locomotor skills despite rapid mortality around four months of age. Given the involvement of the heart and nervous system in Farber disease, the rapid loss in DKO fish survival could be due to sudden decline in organ function such as heart failure or fatal seizures; close monitoring of the DKO population and additional phenotypic characterisations could help uncover the cause of early death and shed light on the mechanisms of disease progression.

In additional to reduced size and lifespan, total brain ceramide content was increased by 15-fold in DKO zebrafish relative to SKO siblings. Given the wide range of ceramide functions, ceramide accumulation likely contributes toward early mortality *via* multiple pathways. Sphingolipids are required for early vertebrate development, and disrupted sphingolipid metabolism can lead to defects in organogenesis^74,75^. Morpholinos against the sphingosine-1-phosphate receptors *s1pr1* and *s1pr2* severely inhibit intersegmental vessel angiogenesis^65^, and delayed epiboly is observed in a maternal zygotic mutant of ceramide synthase 2b^41^. Zebrafish carrying a nonsense mutation in 3-ketodihydrosphingosine reductase, an enzyme of the *de novo* ceramide biosynthetic pathway, exhibits hepatosplenomegaly that progresses to steatosis and liver injury^66^. While our analysis has not been implemented in DKO and SKO larvae, the stable genetic mutations present in our model suggest that ceramide accumulation may begin in early development, resulting in defects in organogenesis that lead to reduced lifespan later in life. Ceramide accumulation may also perturb homeostasis by shifting cell fate toward apoptosis; in the mitochondrial apoptotic pathway, ceramides form channels in the mitochondrial outer membrane that cooperate with the proapoptotic proteins BAX and BAK to trigger membrane permeabilization^78^. Given the position of ceramide at the centre of the sphingolipid metabolic pathway, drastic alterations in ceramide levels could also translate to changes in additional sphingolipids such as sphingomyelins and hexosylceramides, all of which are critical components of lipid rafts and the myelin sheath^79,80^. Perturbations in either the level or acyl chain distribution of these sphingolipids may negatively impact myelin stability, thus contributing to potential pathologies within the central nervous system.

Surprisingly, the extent of ceramide accumulations present in the DKO zebrafish depended on both LCB and acyl chain lengths; ceramides with longer LCBs were preferentially elevated, while a significantly smaller degree of ceramide accumulation occurred for all species with acyl chains longer than C21. Within the cell, multiple pathways are in place for ceramide generation^40^. While variations in LCB lengths are the consequence of different SPT subunit combinations^68,69^, diversity of acyl chain lengths is driven by ceramide synthases that catalyse the acylation of sphinganine to dihydroceramide^81^. The six known mammalian ceramide synthases (CERS1-6) exhibit distinct tissue localisations and acyl chain preferences^81^. Nine zebrafish Cers orthologues have been identified, with unique expression patterns during embryogenesis, and amino acid identities of 49–80% relative to human CERSs (Table S5)^55,82^. Given the involvement of multiple enzymes and pathways toward ceramide generation, loss of ceramidase activity may trigger multiple compensatory pathways in an effort to normalise ceramide levels. Selective downregulation of Spt heterotrimers and/or ceramide synthases in the absence of Asah1 may lead to the LCB- and acyl chain-specific ceramide changes observed in our zebrafish model.

The rapid reduction in ceramide accumulation that occurs past C21 could also be an indication of altered peroxisomal or mitochondrial function. During fatty acid β-oxidation in mammals, very long chain fatty acids (VLCFAs), typically C22-C26, are transported to the peroxisome, where they are oxidized to shorter-chain species that are trafficked to the mitochondria for further breakdown^83^. Short- to medium-chain FAs are typically catabolized in the mitochondria without peroxisomal involvement^84^. Loss-of-function mutations in the VLCFA transporter ABCD1 leads to X-linked adrenoleukodystrophy (ALD), one of the most frequently occurring peroxisomal disorders that involve progressive loss of myelin and early mortality; elevated VLCFA is detected in the blood of 99% of male patients, and is one of the diagnostic tools for ALD^85^. Given the function of the peroxisome in metabolite clearance, significant ceramide storage at the lysosome could trigger increased peroxisomal metabolism as a compensatory mechanism, thereby reducing the availability of the VLCFA pool for ceramide generation. An expansion of our PRM method toward other lipid families could help address the question of additional organelle involvement, as well as the roles of additional lipids in disease progression.

## Methods

### Materials

d18:1/16:0 (860516), d18:1/17:0 (860517), d18:1-d_7_/15:0 (860681), d18:1-d_7_/18:0 (860677) and d18:1-d_7_/24:1 (860679) ceramides were purchased from Avanti Polar Lipids. Water (4218-03) was from J.T. Baker. Methanol (MX0486-1) and isopropanol (PX1834-1) were from Sigma-Aldrich. Ammonium formate (A1190) was from Spectrum. Formic acid (28905) was from ThermoFisher Scientific. Guard cartridge (6956) and analytical column (84410) for LC–MS were from Dikma. Cas9 nuclease (M0386M) was from New England Biolabs. Proteinase K (03115828001) was from Roche. PCR reagents were from New England Biolabs and Promega. Reagents for cloning were from Promega and ThermoFisher Scientific. DNA and RNA kits were from Qiagen and ThermoFisher Scientific. Bradford assay reagent was from Bio-Rad. Glassware for lipid extraction was from VWR. Oligos were synthesised at the Massachusetts General Hospital Center for Computational & Integrative Biology DNA Core, the University of Utah DNA Sequencing Core Facility, or IDT.

### Animals

All zebrafish husbandry and experiment protocols were approved by and carried out in accordance with the Institutional Animal Care and Use Committee at Massachusetts General Hospital or University of Utah.

### Sample collection

HEK293 cells were maintained at 37 °C and 5% CO_2_ in Dulbecco’s Modified Eagle Medium (high glucose) supplemented with 10% foetal bovine serum, penicillin and streptomycin. Cells were grown in 100 mm dishes, passaged at least three times and harvested at confluency (48–52 hours after last passage) by scraping. Zebrafish embryos and larvae were euthanised by cooling the Petri dish on ice, and the euthanised embryos or larvae transferred into Falcon tubes by pipetting. Adult TuAB zebrafish (12 months of age for all wild-type fish, 3.5 months for DKO and SKO fish due to early mortality in the former) were euthanised by immersion in ice-chilled water, following which the head was removed and the brain rapidly excised and flash frozen in liquid nitrogen prior to storage at −80 °C.

### Lipid extraction

All samples were extracted using a modified version of the Bligh-Dyer method^86^. Briefly, one adult zebrafish brain or 35–150 zebrafish larvae were homogenised 40 times on ice with a 7 mL glass dounce homogeniser (VWR, KT885300-0007) with pestle A in a mixture of 1.5 mL aqueous buffer (100 mM trisodium citrate, 1 M sodium chloride, pH 3.6), 1.5 mL methanol, and 3.0 mL chloroform containing internal standards. The resulting mixture was transferred into a glass vial with PTFE-lined cap (VWR, 66009-984), vortexed for 15 s, and centrifuged at 2000 g for 8 min to induce phase separation. The organic layer was retrieved with a Pasteur pipette, dried under a gentle stream of nitrogen and stored at −80 °C. HEK293 cells (~10^7^ cells/sample) were homogenized by manual shaking for 30 s in glass vials with PTFE-lined caps, in the same volume of aqueous-organic mixture as zebrafish samples; subsequent steps were identical to zebrafish samples. All samples were analysed within two weeks. 1/6–1/4 of each sample was used for analysis. Protein concentrations for all samples were determined by Bradford assay.

### Lipidomics

Parallel Reaction Monitoring was performed in positive ionisation mode on an Ultimate 3000 HPLC online with a Thermo q-Exactive Plus quadrupole-orbitrap mass spectrometer equipped with a heated electrospray ion source. Solvent A was 95: 5 water: methanol, 5 mM ammonium formate, 0.1% formic acid. Solvent B was 60: 35: 5 isopropanol: methanol: water, 5 mM ammonium formate, 0.1% formic acid. A Bio-Bond C4 column (Dikma, 5 µm, 50 × 4.6 mm) coupled to a Bio-Bond C4 guard cartridge (Dikma, 5 µm, 10 × 4.0 mm) was used. The gradient was held at 0% B between 0 and 5 min, raised to 20% B at 5.1 min, increased linearly from 20% to 100% B between 5.1 and 55 min, held at 100% B between 55 min and 63 min, returned to 0% B at 63.1 min, and held at 0% B until 70 min. Flow rate was 0.1 mL/min from 0 to 5 min, 0.4 mL/min between 5.1 min and 55 min, and 0.5 mL/min between 55.1 min and 70 min. Divert valve was set to waste for 1–1.5 min and 62–70 min, and to MS for rest of run. Spray voltage was 4.0 kV. Sheath, auxiliary and spare gases were 52.5, 13.75 and 2.75, respectively. Capillary temperature was 268.75 °C. S-lens RF level was 50. MS^2^ was acquired with a resolution of 35000, target ion 2e5, maximum injection time 100 ms and isolation window 1.0 *m/z*. Stepped normalised collision energies were 20, 30. The *m/z* inclusion list is provided in Table S1. All data analyses were performed by manual peak integration in Thermo Xcalibur. Three internal standards were used for quantification. C32–C34, C35–C39 and C40–C44 ceramides were quantified using the d18:1-d_7_/15:0, d18:1-d_7_/18:0 and d18:1-d_7_/24:1 standards, respectively. Additional notes on chromatography are provided under Supplemental Discussion and Figs. S6–S10.

### Generation of *asah1a/b*^−/−^ zebrafish *via* CRISPR-Cas9 mutagenesis

#### Guide generation

CRISPR guides were designed following the method of Gagnon *et al*.^10^. Briefly, three 23-bp sequences targeting exon 10 of zebrafish *asah1a*, and five 23-bp sequences targeting exon 10 of zebrafish *asah1b* were designed with the ChopChop software (https://chopchop.cbu.uib.no) using NGG as the PAM motif. The final gene-specific oligo was 5′-ATTTAGGTGACACTATA(N_20_)GTTTTAGAGCTAGAAATAGCAAG-3′, where N_20_ refers to the 20-bp (excluding PAM) gene-specific targeting sequence, which is preceded by the 17-bp SP6 promoter sequence and followed by a 23-bp sequence that overlaps with a 80-bp constant region required for Cas9 recogniti on. The constant region sequence was 5′-AAAAGCACCGACTCGGTGCCACTTTTTCAAGTTGATA ACGGACTAGCCTTATTTTAACTTGCTATTTCTAGCTCTAAAAC-3′. N_20_ sequences were 5′-AGGAATTCTCACAGGGATCC-3′, 5′-GAGTTCTGGAAAATTCTACC-3′ and 5′-AGGCTGTTGTTGGTTTTACC-3′ for *asah1a*; and 5′-GAATTACAGGGATTCTGGAG-3′, 5′-TGGATCTTGGGAAAGAGAGA-3′, 5′-GGTGTGAATTACAGGGATTC-3′, 5′-AGGGATTCTGGAGTGGATCT-3′ and 5′-GGGATTCTGGAGTGGATCTT-3′ for *asah1b*. DNA template for *in vitro* transcription was generated by PCR following the method of Shah *et al*.^87^.

PCR products were purified using the QIAquick PCR Purification kit (Qiagen, 28104), and immediately used for *in vitro* transcription with the MEGAscript SP6 Transcription kit (ThermoFisher Scientific, AM1330) following the method of Gagnon *et al*.^10^. All reactions (10 μL/reaction) were incubated overnight at 37 °C and purified using RNA Clean & Concentrator kit (Zymo Research, R1013). Yield per guide was 250–1100 ng/μL. All guides were diluted to 200–500 ng/μL in nuclease-free water based on RNA concentration, aliquoted and stored at −80 °C.

#### Guide delivery

Frozen guides (eight guides total, 200–500 ng/μL, 2 μL aliquot per guide) were thawed on ice and 1 μL of each guide was removed and combined. Cas9 nuclease (10% of combined guide volume) was added to the combined guides (final Cas9 concentration ~2 μM), and the resulting solution incubated at room temperature for 10 min. Phenol Red solution (5% of Cas9 and combined guide volume) was added to improve visualisation during microinjection. ~1–2 nL of the guide-Cas9 solution was microinjected into each zebrafish embryo (TuAB strain) at the 1-cell stage.

#### Genotyping and maintenance

F0 zebrafish from injected embryos were in-crossed to obtain F1 fish. Under anaesthesia, a small portion of the tail fin was removed from each F1 fish, digested with proteinase K at 56 °C overnight, and amplified with gene-specific primers for DNA fragment analysis. Based on fragment analysis results, F1 fish carrying deletions and/or insertions were Sanger sequenced, and fish with mutations of interest (i.e. nonsense, frameshift) were selected and individually out-crossed with wild-type fish to yield the heterozygous F2 population, from which subsequent in-crosses (Fig. 4a) yielded the double knockout (DKO) and single knockout (SKO) populations used in the current study. Fragment analysis and Sanger sequencing data were analysed with Geneious and 4Peaks, and all sequence alignments were performed in BLAST^55^. PCR parameters (Promega Gotaq) are 95 °C, 2 min; 36 cycles of 95 °C for 30 s, 56 °C for 30 s, and 72 °C for 45 s; 72 °C, 5 min. *asah1a* forward primer: 5′-ATCATGGGTGCAACTAGATGTG-3′, *asah1a* reverse primer: 5′-AAAAACAGCTTTGCATTGTTCA-3′; *asah1b* forward primer: 5′-CCCTTTATGTTGAATTTGAGGC-3′, *asah1b* reverse primer: 5′-AAACCTAGTTGCATTCTCCAGC-3′. 5′6-FAM was added to all forward primers for DNA fragment analysis. For Sanger sequencing, PCR product was purified using the QIAquick PCR Purification Kit (Qiagen, 28104). The purified PCR product was inserted into the pGEM-T Easy vector (Promega, A1360) or the pCR4-TOPO TA vector (ThermoFisher Scientific, 450030), and transformed into competent cells (New England Biolabs, C3040I). Sanger sequencing was conducted using M13 forward or M13 reverse primer for the pGEM system, and T3 primer for the TOPO system.

For Sanger sequencing of full-length *asah1a* and *asah1b* cDNA, RNA was isolated from *asah1a*(+68/+68), *asah1b*(−20/+) and *asah1a*(+68/+), *asah1b*(−20/−20) zebrafish brains using the RNeasy Lipid Tissue Mini Kit (Qiagen, 74804, one brain per sample). The resulting RNA was diluted to 200 ng/µL, and reversed transcribed (10 µL RNA, 20 µL reaction volume with RNase inhibitor) using the Applied Biosystems High Capacity cDNA Reverse Transcription Kit (ThermoFisher Scientific, 4368814) following manufacturer’s instructions. The full cDNA sequences of *asah1a* and *asah1b* were amplified by PCR. PCR parameters (Promega Gotaq) were 95 °C, 2 min; 36 cycles of 95 °C for 30 s, 56.1 °C for 30 s (*asah1a*) or 60.7 °C for 30 s (*asah1b*), and 72 °C for 1 min 15 s; 72 °C, 5 min. *asah1a* forward primer: 5′-GATGAAGCTTGTGTTCCGTTAC-3′, *asah1a* reverse primer: 5′-TCGGAGTTGATGGCAGATTAC-3′; *asah1b* forward primer: 5′-CATGAACAACAGATTAAACCTG-3′, *asah1b* reverse primer: 5′-CAGAAGTGTACTATGGTCTTGAG-3′. PCR product was purified using the QIAquick PCR Purification Kit (Qiagen, 28104). The purified PCR product was inserted into the pCR4-TOPO TA vector (ThermoFisher Scientific, 450030) and transformed into competent cells (New England Biolabs, C3040I). Sanger sequencing was conducted using both M13 forward and M13 reverse primers. DNA fragment analysis and Sanger sequencing were performed at the Massachusetts General Hospital Center for Computational & Integrative Biology; the Genomics Core Facility, a part of the Health Sciences Cores at the University of Utah; or Genewiz.

## Supplementary information

Supplementary Information

## Data Availability

The datasets and model organism generated in this study are available from the corresponding author on reasonable request.
