# Supplementary material for: Parallel Reaction Monitoring reveals structure-specific ceramide alterations in the zebrafish
Source: Sci Rep. 2019 Dec 27;9:19939. doi: 10.1038/s41598-019-56466-z (PMC6934720; doi:10.1038/s41598-019-56466-z)
Supplement: Supplementary file 1 — Supplementary Information [file 41598_2019_56466_MOESM1_ESM.pdf]

SUPPLEMENTAL INFORMATION

Parallel Reaction Monitoring reveals structure-specific ceramide alterations in the zebrafish

Tejia Zhang[1], Sunia A. Trauger[2], Charles Vidoudez[2], Kim P. Doane[1], Brock R. Pluimer,[1] Randall T. Peterson[1,*]

[1]Department of Pharmacology and Toxicology, College of Pharmacy, University of Utah, Salt Lake City, Utah, USA

[2]Small Molecule Mass Spectrometry, Harvard University, Cambridge, Massachusetts, USA

*Corresponding author, email: randall.peterson@pharm.utah.edu

The following materials are included under Supplemental Information:

Supplemental Methods

Supplemental Tables and Figures: **Tables S1-5**, **Figs. S1-S10**

Supplemental Discussion: Additional notes on chromatography (**Figs. S6-S10**)

SUPPLEMENTAL METHODS

Lipidomics (Fig. 3, Fig. S8b, Fig. S10b)

In addition to targeted ceramide detection, untargeted lipidomic analysis was also performed on adult zebrafish brains on the same LC–MS system, using identical LC conditions as PRM mode. Data were acquired in full MS/DD–MS^2 (top5) mode. Spray voltage was 4.0 kV. Sheath, auxiliary and spare gases were 55.0, 14.00 and 3.00, respectively. Capillary temperature was 269.00 °C. S-lens RF level was 50. MS^1 was acquired with a resolution of 70000, target ion 1e6 and maximum injection time 100 ms. MS^2 was acquired with a resolution of 35000, target ion 1e5, maximum injection time 70 ms and isolation window 1.0 *m/z*. Stepped normalised collision energies were 20, 30. All data analyses were performed by manual peak integration in Thermo Xcalibur.

Visceral organ and oocyte isolation

Adult female zebrafish were euthanised by immersion in water precooled with ice, and the visceral organs (all visceral organs with the exception of kidney, which was left attached to the dorsal wall) were removed from the abdominal cavity. The oocytes were separated from additional visceral mass, and both fractions were transferred into Eppendorf tubes, immediately flash frozen in liquid nitrogen and stored at -80 ºC. Lipid extraction was identical to brain samples.

SUPPLEMENTAL TABLE AND FIGURES

Table S1. PRM inclusion list.

Compound name	Mass [*m/z*]	CS	Polarity	NCE
C32:2 ceramide	508.4724	1	positive	20, 30
C32:1 ceramide	510.4881	1	positive	20, 30
C33:2 ceramide	522.4881	1	positive	20, 30
C33:1 ceramide	524.5037	1	positive	20, 30
C34:2 ceramide	536.5037	1	positive	20, 30
C34:1 ceramide	538.5194	1	positive	20, 30
C35:2 ceramide	550.5194	1	positive	20, 30
C35:1 ceramide	552.5350	1	positive	20, 30
C36:2 ceramide	564.5350	1	positive	20, 30
C36:1 ceramide	566.5507	1	positive	20, 30
C37:2 ceramide	578.5507	1	positive	20, 30
C37:1 ceramide	580.5663	1	positive	20, 30
C38:2 ceramide	592.5663	1	positive	20, 30
C38:1 ceramide	594.5820	1	positive	20, 30
C39:2 ceramide	606.5820	1	positive	20, 30
C39:1 ceramide	608.5976	1	positive	20, 30
C40:2 ceramide	620.5976	1	positive	20, 30
C40:1 ceramide	622.6133	1	positive	20, 30
C41:2 ceramide	634.6133	1	positive	20, 30
C41:1 ceramide	636.6289	1	positive	20, 30
C42:3 ceramide	646.6133	1	positive	20, 30
C42:2 ceramide	648.6289	1	positive	20, 30
C42:1 ceramide	650.6446	1	positive	20, 30
C43:2 ceramide	662.6446	1	positive	20, 30
C43:1 ceramide	664.6602	1	positive	20, 30
C44:2 ceramide	676.6602	1	positive	20, 30
C44:1 ceramide	678.6759	1	positive	20, 30
C45:2 ceramide	690.6759	1	positive	20, 30
C45:1 ceramide	692.6915	1	positive	20, 30
C46:2 ceramide	704.6915	1	positive	20, 30
C46:1 ceramide	706.7072	1	positive	20, 30
C47:2 ceramide	718.7072	1	positive	20, 30
C47:1 ceramide	720.7228	1	positive	20, 30
C48:2 ceramide	732.7228	1	positive	20, 30
C48:1 ceramide	734.7385	1	positive	20, 30
d18:1-d_7/15:0 ceramide	531.5477	1	positive	20, 30
d18:1-d_7/24:1 ceramide	655.6729	1	positive	20, 30
d18:1-d_7/18:0 ceramide	573.5946	1	positive	20, 30

CS: charge state, NCE: normalised collision energy.

Table S2. Ceramide levels (pmol/mg protein) across zebrafish brain, larva and HEK293 sample replicates, related to Table 1

Ceramide	Isomer	Brain_1	Brain_2	Brain_3	Brain_4	SD	Larvae_1	Larvae_2	Larvae_3	Larvae_4	SD	HEK293_1	HEK293_2	HEK293_3	HEK293_4	SD
C32:2	d18:2/14:0											2.9	3.7	4.9	3.8	0.8
C32:1	d16:1/16:0						2.7	3.2	2.4	2.4	0.4	7.1	8.1	8.2	8.7	0.7
	d18:1/14:0											14.5	16.9	17.2	18.2	1.6
C33:1	d17:1/16:0						7.1	8.4	6.8	6.4	0.9	9.3	10.7	11.1	11.9	1.1
	d18:1/15:0						0.7	0.8	0.7	0.6	0.1	0.7	0.8	0.8	0.9	0.1
C34:2	d18:2/16:0	0.0	7.4	2.8	8.9	4.1						39.6	47.4	66.3	51.3	11.2
	d18:1/16:1						6.9	8.2	7.8	6.8	0.7	3.9	4.9	6.3	5.0	1.0
C34:1	d16:1/18:0											2.2	2.4	2.6	2.9	0.3
	d18:1/16:0	79.6	155.1	79.2	138.4	39.5	309.4	343.6	267.4	263.6	37.9	310.2	348.8	377.2	398.1	38.0
C35:2	d17:1/18:1						2.1	2.6	2.9	2.4	0.3					
	d18:2/17:0											1.2	1.6	2.3	1.7	0.5
	d19:1/16:1						1.3	1.6	1.5	1.2	0.2					
C35:1	d17:1/18:0	12.9	10.1	9.7	12.4	1.6	5.0	5.8	5.0	4.3	0.6	5.0	5.7	5.9	6.7	0.7
	d18:1/17:0	6.3	9.6	8.2	11.7	2.3	7.3	9.0	6.3	6.0	1.4	8.2	10.3	10.6	11.2	1.3
	d19:1/16:0						7.4	9.1	6.8	6.3	1.2	1.9	2.5	2.7	2.5	0.3
C36:2	d18:2/18:0	0.0	7.7	3.7	9.0	4.1						40.9	51.2	73.4	53.0	13.6
	d18:1/18:1	12.3	21.2	27.9	30.8	8.2	4.9	6.0	6.2	5.9	0.6	5.4	6.9	8.7	7.3	1.4
C36:1	d16:1/20:0						0.7	0.8	0.6	0.6	0.1	1.4	1.8	2.0	2.3	0.4
	d18:1/18:0	470.9	571.6	532.7	700.2	96.9	150.6	171.8	133.4	123.8	21.1	52.0	60.5	63.8	70.0	7.5
C37:1	d17:1/20:0						2.4	2.8	2.2	2.1	0.3	0.9	0.9	0.8	1.1	0.1
	d18:1/19:0	9.8	12.3	13.6	12.3	1.6	7.5	9.4	7.4	6.6	1.2	0.7	1.0	1.1	1.3	0.2
	d19:1/18:0	10.2	13.8	12.0	17.0	2.9	4.0	4.9	4.1	3.5	0.6	1.0	1.1	1.1	1.3	0.2
C38:2	d16:1/22:1	4.4	3.1	4.2	3.8	0.6						0.8	0.9	1.2	1.1	0.2
	d18:2/20:0											7.4	8.5	13.5	9.4	2.6
	d18:1/20:1	8.3	11.0	20.5	19.5	6.1	2.9	3.1	3.2	3.4	0.2					
C38:1	d16:1/22:0	4.8	2.8	3.0	4.6	1.1	8.8	10.1	7.9	6.5	1.5	12.7	14.9	14.8	16.6	1.6
	d18:1/20:0	58.3	65.2	82.5	74.8	10.7	111.9	141.3	110.6	86.6	22.4	30.7	36.4	35.6	42.3	4.7
	d20:1/18:0	86.1	80.0	104.6	120.7	18.5	12.5	14.9	12.0	8.8	2.5	1.5	1.7	1.7	1.8	0.1
C39:1	d16:1/23:0						4.5	5.7	5.9	4.7	0.7	4.5	5.1	6.6	5.7	0.9
	d17:1/22:0	13.0	10.3	13.9	13.9	1.7	28.6	37.6	35.6	30.0	4.4	13.0	16.2	21.4	17.9	3.5
	d18:1/21:0	8.0	8.5	13.9	13.7	3.2	15.9	21.0	19.8	16.0	2.6	2.7	3.1	4.0	3.4	0.5
	d19:1/20:0						3.5	4.8	4.4	3.7	0.6					
	d20:1/19:0						0.7	1.1	1.0	0.8	0.2					
C40:2	d16:1/24:1	10.8	6.2	6.2	7.2	2.2	2.6	2.9	2.4	2.3	0.3	3.1	3.1	3.5	3.6	0.3
	d18:2/22:0											19.1	19.1	21.6	22.1	1.6
	d18:1/22:1	140.5	125.8	200.9	201.8	39.8	5.4	5.4	4.8	4.5	0.4	5.4	5.4	5.8	6.1	0.3
C40:1	d16:1/24:0	5.1	4.3	3.6	5.4	0.8	3.4	3.8	3.5	3.1	0.3	13.4	14.1	15.9	16.1	1.4
	d17:1/23:0						1.8	2.1	1.8	1.5	0.2	0.6	0.7	0.8	0.8	0.1
	d18:1/22:0	213.4	164.6	265.0	272.2	50.2	73.6	84.5	74.3	65.0	8.0	47.7	52.7	60.4	59.6	6.0
	d20:1/20:0	2.9	2.9	3.9	3.4	0.5	1.4	1.7	1.4	1.3	0.2					
C41:2	d17:2/24:0											0.4	0.4	0.4	0.4	0.0
	d17:1/24:1	18.3	11.7	15.4	14.5	2.7	7.0	8.0	6.7	6.1	0.8	4.5	4.6	4.9	5.3	0.4
	d18:2/23:0											4.4	4.5	5.2	5.2	0.4
	d18:1/23:1	36.5	34.4	51.7	52.2	9.6	2.3	2.5	2.0	1.9	0.3	2.9	2.8	3.1	3.2	0.2
C41:1	d16:1/25:0						0.5	0.6	0.5	0.4	0.0					
	d17:1/24:0	5.6	5.5	5.1	6.1	0.4	6.6	7.1	6.9	6.2	0.4	10.3	12.0	12.8	13.4	1.3
	d18:1/23:0	41.8	47.2	53.7	59.9	7.9	18.1	19.1	17.7	16.5	1.1	19.5	21.2	23.1	23.9	2.0
	d19:1/22:0						1.7	1.9	1.6	1.6	0.2	0.5	0.5	0.6	0.6	0.1
C42:2	d16:1/26:1						0.6	0.8	0.7	0.6	0.1					
	d18:2/24:0	0.4	10.9	6.4	13.6	5.8						107.0	113.0	119.8	127.4	8.8
	d18:1/24:1	827.8	766.7	921.2	981.0	95.4	60.1	66.0	57.2	53.2	5.4	90.9	95.6	111.7	112.8	11.1
C42:1	d16:1/26:0						1.4	1.7	1.4	1.3	0.2	0.3	0.3	0.3	0.3	0.0
	d17:1/25:0						1.1	1.3	1.1	0.9	0.2					
	d18:1/24:0	332.4	354.8	312.9	463.1	67.1	85.0	95.1	76.9	70.4	10.7	213.4	225.6	235.3	276.3	27.3
	d19:1/23:0						0.5	0.6	0.5	0.5	0.1					
	d20:1/22:0											0.4	0.4	0.5	0.6	0.1
C43:2	d17:1/26:1						1.2	1.6	1.4	1.3	0.2					
	d18:2/25:0											1.7	1.9	2.2	2.2	0.2
	d18:1/25:1	22.5	24.5	26.7	28.8	2.7	3.4	4.1	3.6	3.2	0.4	1.7	1.8	1.9	2.0	0.1
	d19:1/24:1	7.1	6.3	7.4	9.1	1.2	1.4	1.6	1.3	1.2	0.1	1.0	1.2	1.3	1.2	0.1
C43:1	d17:1/26:0						2.5	2.9	2.5	2.2	0.3					
	d18:1/25:0	9.6	13.9	9.6	14.6	2.7	7.9	9.1	7.8	7.2	0.8	4.9	5.3	5.6	6.0	0.5
	d19:1/24:0						2.2	2.3	1.9	1.8	0.2	1.6	1.9	2.0	2.1	0.2
C44:2	d18:2/26:0											2.3	2.5	2.7	2.8	0.2
	d18:1/26:1	127.3	135.8	144.7	182.4	24.3	13.1	15.2	13.7	11.4	1.6	3.3	3.3	3.8	3.9	0.3
	d20:2/24:0											0.5	0.5	0.6	0.6	0.0
	d20:1/24:1	10.3	8.4	10.7	13.1	2.0						1.0	1.0	1.2	1.1	0.1
C44:1	d18:1/26:0	34.9	49.1	32.4	56.9	11.7	21.2	26.1	21.9	19.3	2.9	4.2	4.7	4.7	5.0	0.3
	d20:1/24:0											1.8	1.8	1.9	2.2	0.2

Table S3. Ceramide levels across 48 hpf, 4 dpf and 7 dpf zebrafish larva sample replicates, related to Fig. 3

LCB	Isomer	48 hpf larvae_1	48 hpf larvae_2	48 hpf larvae_3	48 hpf larvae_4	SD	4 dpf larvae_1	4 dpf larvae_2	4 dpf larvae_3	4 dpf larvae_4	SD	7 dpf larvae_1	7 dpf larvae_2	7 dpf larvae_3	7 dpf larvae_4	SD
	d16:1/16:1	3.4	3.0	2.6	2.5	0.4	2.3	3.0	2.3	3.1	0.4	1.7	1.3	1.7	1.4	0.2
	d16:1/16:0	28.4	26.7	24.7	20.6	3.3	24.9	35.3	29.9	43.8	8.1	14.7	13.4	15.8	12.2	1.6
	d16:1/20:0											1.5	1.2	1.6	1.9	0.3
	d16:1/22:0	4.3	4.6	4.6	5.7	0.6	6.6	6.2	5.0	11.1	2.6	5.1	4.3	7.3	8.8	2.1
d16:1	d16:1/23:0	2.3	2.4	2.9	3.4	0.5	3.4	3.2	2.0	6.0	1.7					
	d16:1/24:1	10.8	9.2	8.0	9.6	1.2	12.3	11.5	10.4	11.3	0.8	9.3	7.2	10.7	10.0	1.5
	d16:1/24:0	4.5	3.8	3.7	4.3	0.4	6.9	6.9	7.0	7.9	0.5	4.3	3.4	5.0	4.7	0.7
	d16:1/25:0	1.2	1.1	1.1	1.3	0.1	1.6	1.4	1.4	2.1	0.3					
	d16:1/26:1	3.8	3.4	3.4	4.0	0.3	4.0	3.5	3.3	4.4	0.5	1.8	1.4	2.6	2.5	0.6
	d16:1/26:0	3.0	2.6	2.5	3.1	0.3	3.6	3.6	3.5	4.4	0.4	1.6	1.2	1.8	1.9	0.3
	d17:1/16:1	1.4	1.0	1.0	1.0	0.2	1.5	1.8	1.4	1.8	0.2					
	d17:1/16:0	5.4	4.7	4.6	3.5	0.8	6.8	8.3	8.1	9.8	1.2	6.5	6.8	6.5	6.3	0.2
	d17:1/18:0						2.6	2.3	1.6	3.0	0.6	2.7	2.3	3.5	3.5	0.6
	d17:1/22:0	2.9	3.0	3.5	4.1	0.5	4.6	4.3	3.3	9.0	2.5	4.4	3.2	5.0	6.9	1.5
d17:1	d17:1/23:0	1.2	1.0	1.0	1.1	0.1	1.8	1.6	1.3	1.8	0.2					
	d17:1/24:1	7.0	6.2	6.1	6.7	0.4	8.4	7.8	6.9	9.2	0.9	7.1	5.7	7.8	8.3	1.2
	d17:1/24:0	3.3	2.6	2.9	3.2	0.3	5.0	4.9	5.0	5.9	0.5	4.0	2.8	4.5	4.2	0.8
	d17:1/25:0	1.0	0.8	0.9	1.0	0.1	1.2	1.2	0.9	1.5	0.2					
	d17:1/26:1	2.3	1.9	2.0	2.4	0.3	2.7	2.2	1.9	2.9	0.4	1.4	1.3	1.8	2.0	0.3
	d17:1/26:0	2.1	1.7	1.7	1.9	0.2	2.5	2.4	2.5	2.9	0.2	1.3	1.1	1.6	1.5	0.2
	d18:1/14:0	4.8	4.4	4.4	3.4	0.6	4.3	6.0	5.5	7.1	1.2	2.2	2.1	2.1	1.8	0.2
	d18:1/15:0						0.9	1.1	1.1	1.3	0.2					
	d18:1/16:1	11.4	9.3	9.3	7.6	1.5	20.8	25.7	21.3	31.1	4.8	26.3	23.5	26.4	21.4	2.4
	d18:1/16:0	38.6	33.1	32.2	33.7	2.9	93.6	85.6	87.3	106.2	9.4	147.8	122.5	175.0	156.6	21.8
	d18:1/17:0	5.3	3.7	3.0	2.6	1.2	9.0	8.1	7.2	8.9	0.8	12.9	10.3	9.5	9.6	1.6
	d18:1/18:0	10.2	9.8	9.7	9.0	0.5	130.3	108.0	96.3	132.4	17.5	144.3	121.5	162.2	160.4	18.9
	d18:1/19:0	0.9	0.7	1.0	0.7	0.1	6.0	5.4	3.9	7.6	1.5	6.7	5.7	7.2	9.6	1.6
	d18:1/20:1						4.4	4.1	5.4	4.8	0.6	8.1	5.9	9.3	7.1	1.5
	d18:1/20:0	24.7	24.1	28.7	29.2	2.7	62.9	55.3	47.8	92.8	19.7	61.3	54.6	69.2	97.7	19.0
d18:1	d18:1/21:0	2.9	3.5	4.2	4.4	0.7	4.2	4.0	2.8	8.6	2.6	4.1	3.8	4.4	7.3	1.6
	d18:1/22:1	4.6	3.9	3.4	3.5	0.5	10.2	8.6	8.7	8.5	0.8	10.4	9.1	11.2	10.8	0.9
	d18:1/22:0	51.4	44.7	43.8	48.5	3.5	73.6	69.5	64.8	86.3	9.2	85.1	65.6	89.1	92.2	11.9
	d18:1/23:1	2.2	2.0	2.0	2.2	0.1	2.8	2.6	2.4	3.2	0.4	2.6	2.1	2.6	3.1	0.4
	d18:1/23:0	27.8	24.0	25.2	28.0	2.0	24.1	23.6	21.5	27.9	2.7	19.3	15.2	20.8	20.7	2.6
	d18:1/24:1	109.7	102.3	98.8	110.0	5.5	137.1	124.1	113.2	145.9	14.4	123.7	97.9	128.5	136.9	16.8
	d18:1/24:0	93.0	79.3	81.1	95.4	8.1	90.5	88.0	87.0	107.9	9.8	74.6	58.2	76.4	77.4	9.0
	d18:1/25:1	10.0	8.4	8.7	10.1	0.9	8.4	8.6	7.8	9.8	0.8	5.9	5.1	6.6	7.0	0.8
	d18:1/25:0	20.2	17.3	18.0	20.0	1.4	14.7	15.4	14.2	16.9	1.2	7.0	6.2	7.8	7.4	0.7
	d18:1/26:1	41.4	36.2	38.4	43.4	3.2	40.5	39.0	38.8	47.9	4.3	27.3	23.6	30.4	29.6	3.1
	d18:1/26:0	39.4	34.0	34.7	39.4	2.9	34.3	33.8	34.4	39.3	2.6	21.3	16.3	23.7	19.8	3.1
	d19:1/16:0	3.6	3.6	4.6	4.7	0.6	7.1	6.5	5.7	9.5	1.6	5.6	4.4	5.4	6.0	0.7
	d19:1/18:0	0.9	0.7	0.9	1.0	0.1	3.6	3.2	1.8	4.2	1.0	2.7	2.6	2.5	3.6	0.5
	d19:1/20:0	2.3	2.0	2.4	3.3	0.6	2.1	1.8	1.0	3.8	1.2					
	d19:1/22:0	8.5	7.1	7.7	8.7	0.7	5.3	5.6	4.5	6.2	0.7	1.7	1.3	1.6	1.6	0.2
d19:1	d19:1/23:0	11.6	10.1	10.4	12.2	1.0	5.4	5.9	5.1	6.2	0.5					
	d19:1/24:1	12.6	11.7	11.8	14.2	1.1	8.1	8.1	8.3	9.4	0.6					
	d19:1/24:0	31.6	27.0	28.9	32.1	2.4	16.0	17.9	15.4	18.1	1.4	1.8	1.6	2.0	1.9	0.2
	d19:1/25:1	3.0	2.7	2.8	3.4	0.3	1.5	1.5	1.3	1.8	0.2					
	d19:1/25:0	6.3	5.6	5.8	6.5	0.4	3.2	3.7	3.0	3.6	0.3					
	d20:1/18:0						4.3	3.0	2.2	5.1	1.3	5.1	5.1	5.6	8.8	1.8
d20:1	d20:1/20:0											1.9	1.5	1.6	2.0	0.2
	d20:1/24:0	1.6	1.3	1.2	1.5	0.2	0.9	0.9	1.3	1.0	0.2					

Table S4. Ceramide levels (pmol/mg protein) across SKO and DKO zebrafish brain sample replicates, related to Fig. 5

LCB	Ceramide	SKO_1	SKO_2	SKO_3	SKO_4	SD	DKO_1	DKO_2	DKO_3	DKO_4	SD	DKO/SKO	ttest
d16:1	d16:1/18:0	1.6	1.3	1.2	0.9	0.3	6.5	10.1	10.7	8.3	1.9	7.0	0.00022
	d16:1/22:0	4.2	2.8	4.5	2.0	1.2	7.2	11.2	8.7	8.4	1.7	2.6	0.00173
	d16:1/24:1	8.0	6.3	6.9	5.0	1.2	16.4	22.5	16.2	16.2	3.1	2.7	0.00053
	d16:1/24:0	2.3	1.7	2.1	1.7	0.3	3.4	4.6	3.3	3.5	0.6	1.9	0.00241
d17:1	d17:1/16:0	2.4	1.4	2.0	1.5	0.5	13.0	13.3	9.8	11.8	1.6	6.6	0.00002
	d17:1/18:1	0.2	0.0	0.2	0.0	0.1	7.7	12.3	8.4	10.9	2.2	120.6	0.00011
	d17:1/18:0	42.4	26.5	29.2	28.5	7.2	175.1	229.7	193.6	233.2	28.2	6.6	0.00002
	d17:1/20:0	0.4	0.1	0.3	0.1	0.2	4.5	5.1	4.1	5.3	0.6	21.5	0.00000
	d17:1/22:1	3.6	1.7	4.3	3.0	1.1	7.6	9.5	6.2	6.3	1.5	2.4	0.00400
	d17:1/22:0	9.5	4.2	6.9	5.4	2.3	19.1	25.1	19.1	25.1	3.5	3.4	0.00029
	d17:1/24:1	11.2	8.7	8.8	8.7	1.2	29.0	39.2	29.6	30.6	4.8	3.4	0.00009
	d17:1/24:0	3.0	2.1	2.7	2.4	0.4	6.9	9.8	7.4	8.1	1.3	3.1	0.00016
	d17:1/26:1	1.6	1.3	1.5	1.4	0.1	5.2	6.4	5.1	5.3	0.6	3.8	0.00001
d18:2	d18:2/18:0	1.8	1.8	1.7	2.7	0.5	19.6	24.2	20.4	36.6	7.8	12.6	0.00104
d18:1	d18:1/14:0	1.1	0.6	1.1	1.0	0.2	9.9	10.9	6.4	12.3	2.5	10.5	0.00038
	d18:1/15:0	0.4	0.4	0.5	0.4	0.0	8.8	8.5	6.2	8.6	1.2	18.9	0.00002
	d18:1/16:1	1.8	1.3	1.3	1.6	0.2	38.4	56.8	36.5	38.2	9.6	28.4	0.00014
	d18:1/16:0	75.5	92.5	68.4	89.7	11.5	2683.4	3864.8	2914.1	2764.3	547.2	37.5	0.00004
	d18:1/17:1	0.2	0.3	0.3	0.3	0.0	8.5	11.7	8.3	11.8	2.0	36.8	0.00006
	d18:1/17:0	20.9	20.1	15.2	18.7	2.5	386.0	553.6	442.3	482.5	70.5	24.9	0.00001
	d18:1/18:1	33.8	22.0	21.3	24.1	5.8	284.5	438.8	379.2	451.6	76.2	15.4	0.00008
	d18:1/18:0	1090.0	929.4	817.0	1026.6	119.1	18223.8	24582.3	21583.0	25775.7	3375.7	23.3	0.00001
	d18:1/19:0	9.2	7.9	5.6	7.6	1.5	212.0	308.4	256.9	272.3	39.9	34.7	0.00001
	d18:1/20:1	19.1	11.4	15.2	17.0	3.2	85.0	138.6	113.2	149.5	28.8	7.8	0.00033
	d18:1/20:0	95.7	65.6	80.4	75.1	12.6	1548.1	2119.1	1838.6	2019.5	250.7	23.7	0.00001
	d18:1/21:0	6.1	5.7	4.8	6.1	0.6	142.0	162.1	127.8	141.4	14.1	25.3	0.00000
	d18:1/22:1	154.5	99.6	122.6	108.2	24.2	296.9	470.4	358.0	371.5	71.9	3.1	0.00055
	d18:1/22:0	247.0	156.8	233.5	148.6	51.0	962.5	1267.8	1039.8	917.6	155.7	5.3	0.00005
	d18:1/23:1	22.8	21.7	19.3	21.6	1.5	52.9	88.9	69.4	62.1	15.3	3.2	0.00087
	d18:1/23:0	23.5	24.3	22.7	26.2	1.5	185.1	248.6	194.9	187.0	30.1	8.4	0.00002
	d18:1/24:1	905.1	671.2	705.7	602.3	130.0	3980.0	5488.5	4780.7	4799.8	616.8	6.6	0.00001
	d18:1/24:0	338.4	277.4	276.3	258.6	34.9	1421.7	2351.6	1921.7	1703.9	392.2	6.4	0.00021
	d18:1/25:1	14.3	13.5	12.2	14.1	0.9	67.4	94.5	81.6	79.3	11.1	6.0	0.00002
	d18:1/25:0	7.7	9.3	6.4	9.6	1.5	64.2	99.6	87.0	78.5	14.9	10.0	0.00006
	d18:1/26:1	127.2	96.3	95.9	100.1	15.0	657.4	910.5	783.8	735.4	106.1	7.4	0.00002
	d18:1/26:0	30.0	29.5	23.6	28.5	3.0	355.7	501.0	402.9	398.5	61.4	14.9	0.00002
d19:1	d19:1/16:0	3.3	4.4	2.6	3.4	0.8	147.1	247.8	139.9	132.2	54.4	48.6	0.00096
	d19:1/18:1	0.0	0.0	0.0	0.0	0.0	5.4	13.0	7.2	7.4	3.3	inf	0.00236
	d19:1/18:0	7.2	10.3	6.1	10.3	2.1	291.6	491.1	379.5	414.7	82.8	46.5	0.00009
	d19:1/19:0	0.0	0.0	0.1	0.0	0.0	10.5	17.3	13.1	13.1	2.8	980.0	0.00007
	d19:1/20:0	0.9	1.3	1.1	1.6	0.3	64.3	98.2	76.6	82.8	14.1	65.2	0.00003
	d19:1/22:1	0.9	0.8	0.7	0.6	0.1	5.7	10.4	6.5	5.3	2.3	9.2	0.00176
	d19:1/22:0	1.0	0.9	0.8	0.8	0.1	24.7	36.0	18.8	16.6	8.7	27.1	0.00175
	d19:1/23:0	0.1	0.2	0.0	0.1	0.1	5.7	7.7	5.4	3.5	1.7	52.6	0.00073
	d19:1/24:1	4.7	5.4	4.2	4.2	0.6	59.2	91.0	62.6	53.2	16.8	14.3	0.00032
	d19:1/24:0	1.6	1.9	1.1	1.5	0.3	19.4	33.3	25.4	20.7	6.3	16.3	0.00032
d20:2	d20:2/18:0	0.0	0.1	0.0	0.2	0.1	8.5	9.2	8.5	14.5	2.9	127.2	0.00041
d20:1	d20:1/17:0	0.0	0.0	0.0	0.0	0.0	3.9	7.1	4.9	6.6	1.5	inf	0.00025
	d20:1/18:0	116.1	100.7	96.7	129.4	15.0	3028.7	3869.6	3545.5	4088.5	460.5	32.8	0.00000
	d20:1/19:0	2.0	1.9	1.3	2.1	0.4	66.1	84.1	76.6	85.7	8.9	42.8	0.00000
	d20:1/20:0	3.5	2.9	3.5	3.2	0.3	77.9	102.0	94.0	87.2	10.2	27.4	0.00000
	d20:1/22:0	2.0	1.0	1.9	1.3	0.5	7.3	9.4	7.1	6.0	1.4	4.7	0.00023
	d20:1/24:1	6.0	3.9	5.3	5.4	0.9	15.8	22.0	19.5	17.8	2.7	3.6	0.00007
	d20:1/24:0	1.6	1.2	1.3	1.3	0.2	6.2	9.0	7.2	6.2	1.3	5.3	0.00013

Table S4. Numbers in red denote ceramide species that fulfilled all four criteria for inclusion (see Results: Ceramide composition in zebrafish and humans) but fell below the linear range of the calibration curves (**Fig. S1**) (calculated pmol on-column value is lower than 0.1). Only ceramides that were within the linear range for both SKO and DKO groups were included in the data for **Fig. 5**; inclusion of the ceramides in red did not significantly alter the reported data.

Table S5. Amino acid identities between human and zebrafish ceramide biosynthesis enzymes[1,2].

Enzyme name	Human	Zebrafish	% AA identity
Serine palmitoyltransferase 1	SPTLC1 (NP_006406)	Sptlc1 (NP_001018307)	85
Serine palmitoyltransferase 2	SPTLC2 (NP_004854)	Sptlc2a (NP_001018455)	82
		Sptlc2b (NP_001108213)	80
Serine palmitoyltransferase 3	SPTLC3 (NP_060797)	Sptlc3 (NP_001003562)	71
Serine palmitoyltransferase small subunit A	SPTSSA (NP_612145)	Sptssa (NP_001087196)	82
Serine palmitoyltransferase small subunit B	SPTSSB (NP_001035189)	Sptssb (NP_001289737)	61
Ceramide synthase 1	CERS1 (NP_001483)	Cers1 (XP_009294228)	55
Ceramide synthase 2	CERS2 (NP_859530)	Cers2a (NP_705957)	65
		Cers2b (XP_693668)	64
Ceramide synthase 3	CERS3 (NP_001277272)	Cers3a (XP_002662790)	52
		Cers3b (NP_001108403)	54
Ceramide synthase 4	CERS4 (NP_078828)	Cers4a (NP_705956)	49
		Cers4b (XP_005163700)	51
Ceramide synthase 5	CERS5 (NP_001317999)	Cers5 (NP_955922)	73
Ceramide synthase 6	CERS6 (NP_001243055)	Cers6 (XP_693283)	80

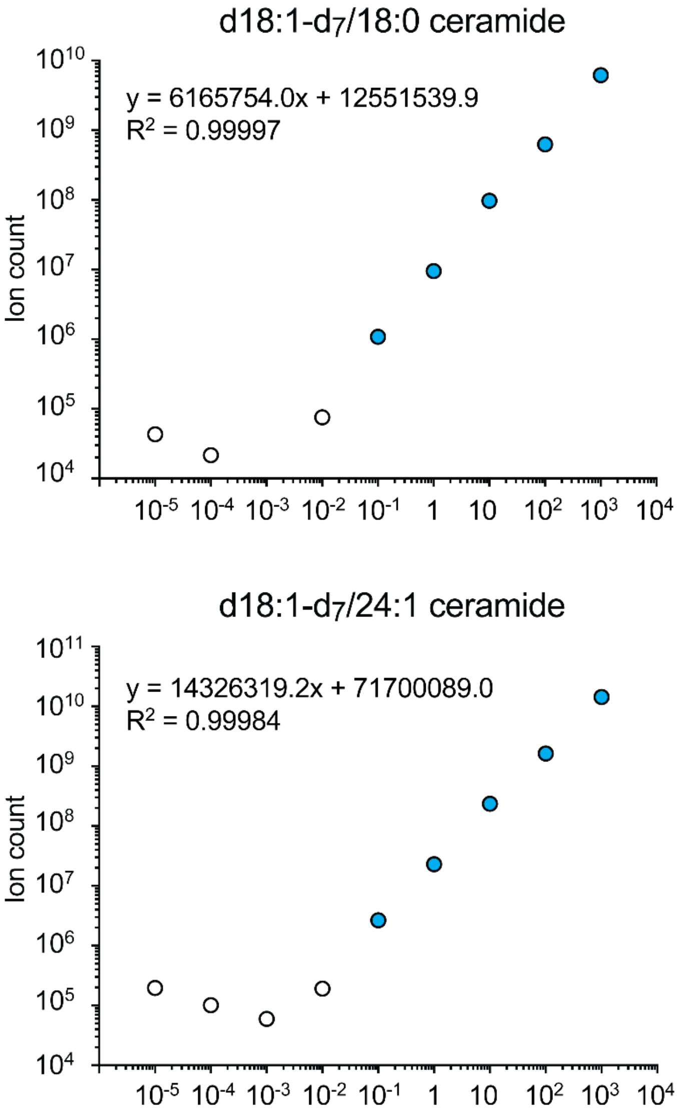

Figure S1. Ceramide calibration curves. Calibration curves for d18:1-d$_7$/18:0 and d18:1-d$_7$/24:1 ceramide standards. Linearity (points in blue) was observed between 100 fmol and 1 nmol of standard on-column.

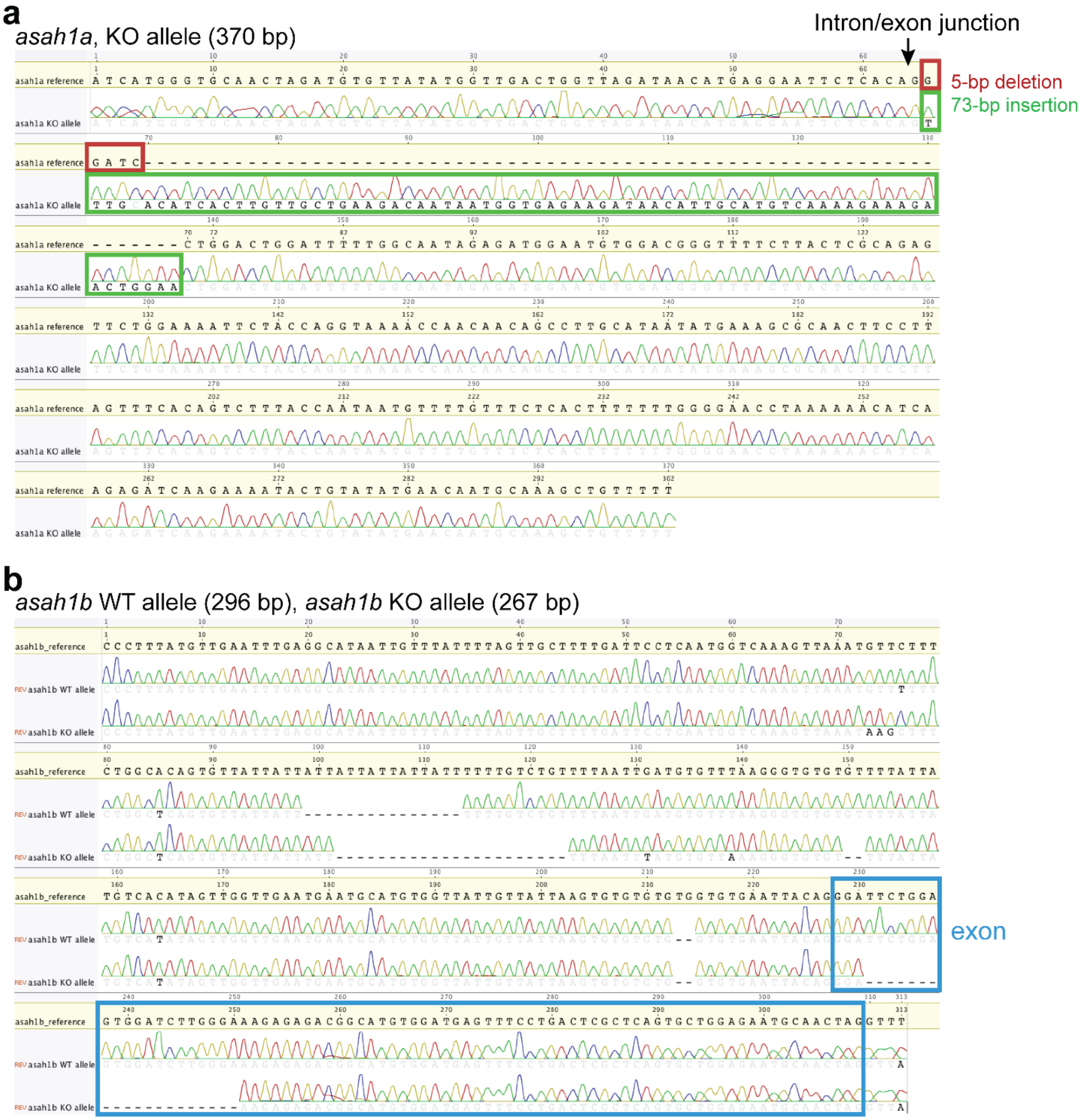

Figure S2. Sanger sequencing of Farber disease zebrafish. (**a**) Alignment of Sanger sequencing results of *asah1a* PCR product from *asah1a*(+68/+68), *asah1b*(-20/+) zebrafish (asah1a KO allele) against reference sequence (asah1a reference: *asah1a* PCR product based on zebrafish genome assembly GRCz11)[3]; asah1a KO allele carries a 5-bp deletion (red box) replaced by a 73-bp insertion (green box). (**b**) Alignment of Sanger sequencing results of *asah1b* PCR products from *asah1a*(+68/+68), *asah1b*(-

20/+) zebrafish (asah1b WT allele, asah1b KO allele) against reference sequence (asah1b reference: *asah1b* PCR product based on zebrafish genome assembly GRCz11)[3]. Boxed region denotes exon. Multiple intronic polymorphisms are present within the WT and KO alleles. Note that the 20-bp deletion in the KO allele may also be shifted one to four nucleotides forward due to presence of the same nucleotide sequence (GGGA) at either end of the deletion. The predicted Cas9 cut site is between position 230 and 231. All alignments were performed in Geneious.

a

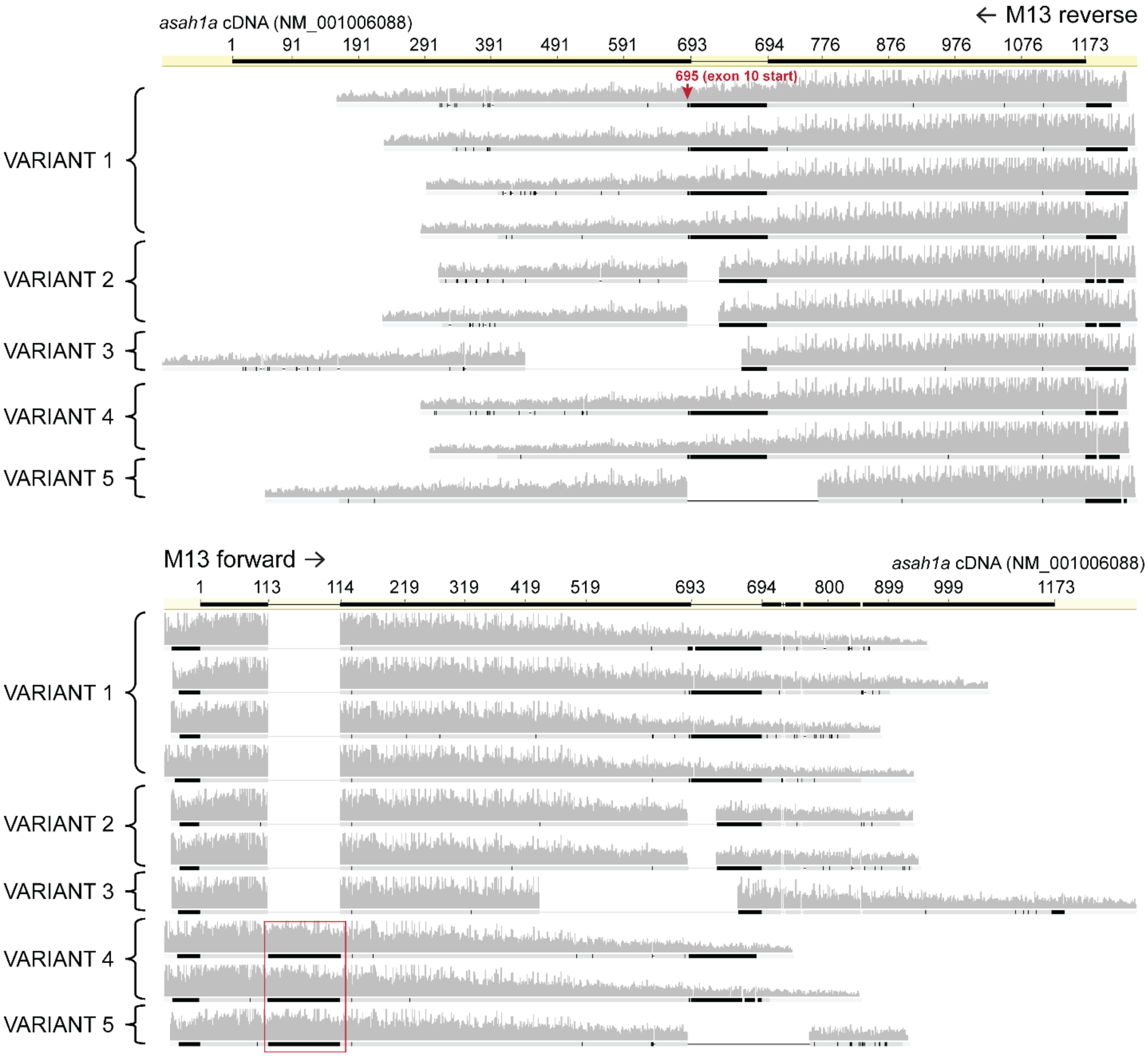
← M13 reverse
asah1a cDNA (NM_001006088)
1 91 191 291 391 491 591 693 694 776 876 976 1076 1173
695 (exon 10 start)
VARIANT 1
VARIANT 2
VARIANT 3
VARIANT 4
VARIANT 5
M13 forward →
asah1a cDNA (NM_001006088)
1 113 114 219 319 419 519 693 694 800 899 999 1173
VARIANT 1
VARIANT 2
VARIANT 3
VARIANT 4
VARIANT 5

b

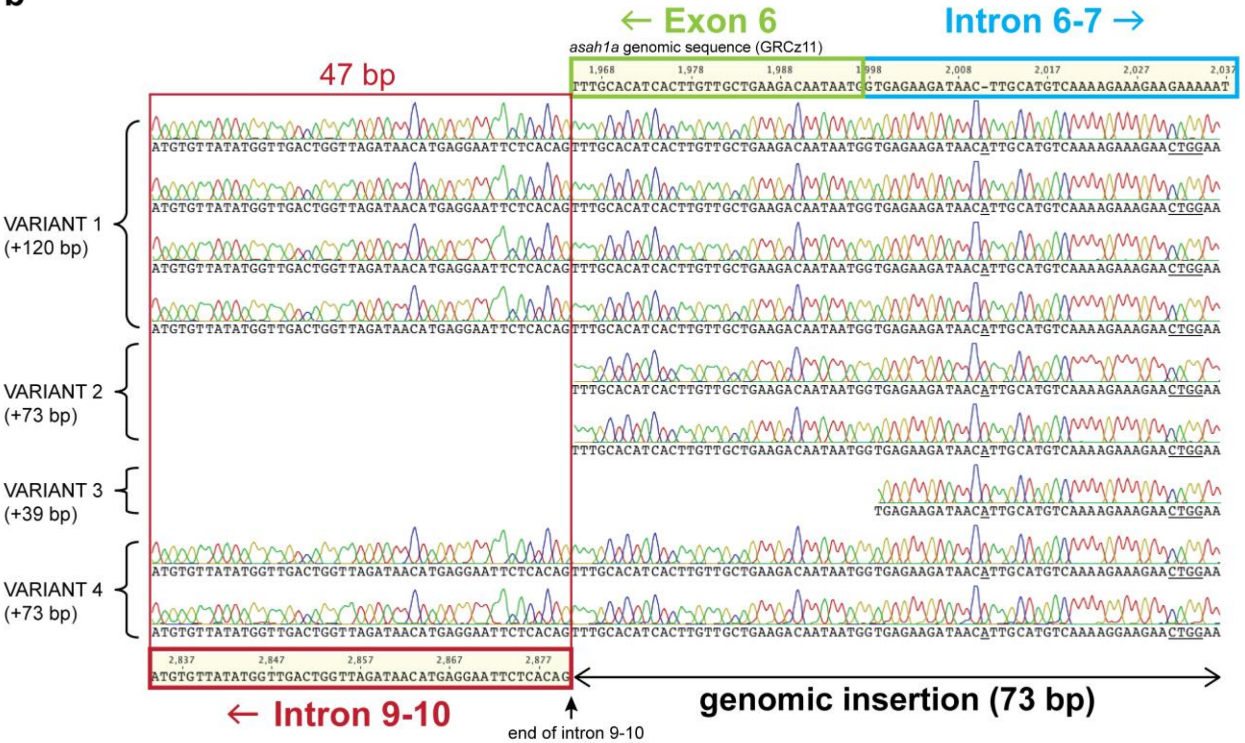
← Exon 6
Intron 6-7 →
asah1a genomic sequence (GRCz11)
47 bp
1,968 1,978 1,988 1,998 2,008 2,017 2,027 2,037
TTTGCACATCACTTGTTGCTGAAGACAATAATGGTGAGAAGATAAC-TTGCATGTCAAAAGAAAGAAGAAAAAT
VARIANT 1
(+120 bp)
VARIANT 2
(+73 bp)
VARIANT 3
(+39 bp)
VARIANT 4
(+73 bp)
2,837 2,847 2,857 2,867 2,877
ATGTGTTATATGGTTGACTGGTTAGATAACATGAGGAATTCTCACAG
← Intron 9-10
end of intron 9-10
genomic insertion (73 bp)

c

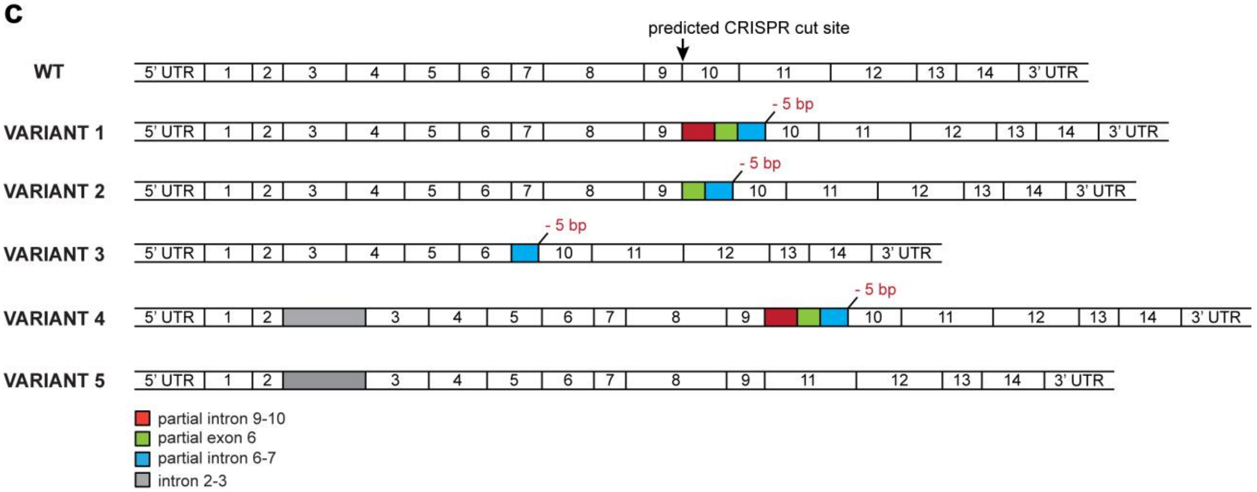
predicted CRISPR cut site
WT
VARIANT 1
VARIANT 2
VARIANT 3
VARIANT 4
VARIANT 5
5' UTR
3' UTR
- 5 bp
partial intron 9-10
partial exon 6
partial intron 6-7
intron 2-3

d

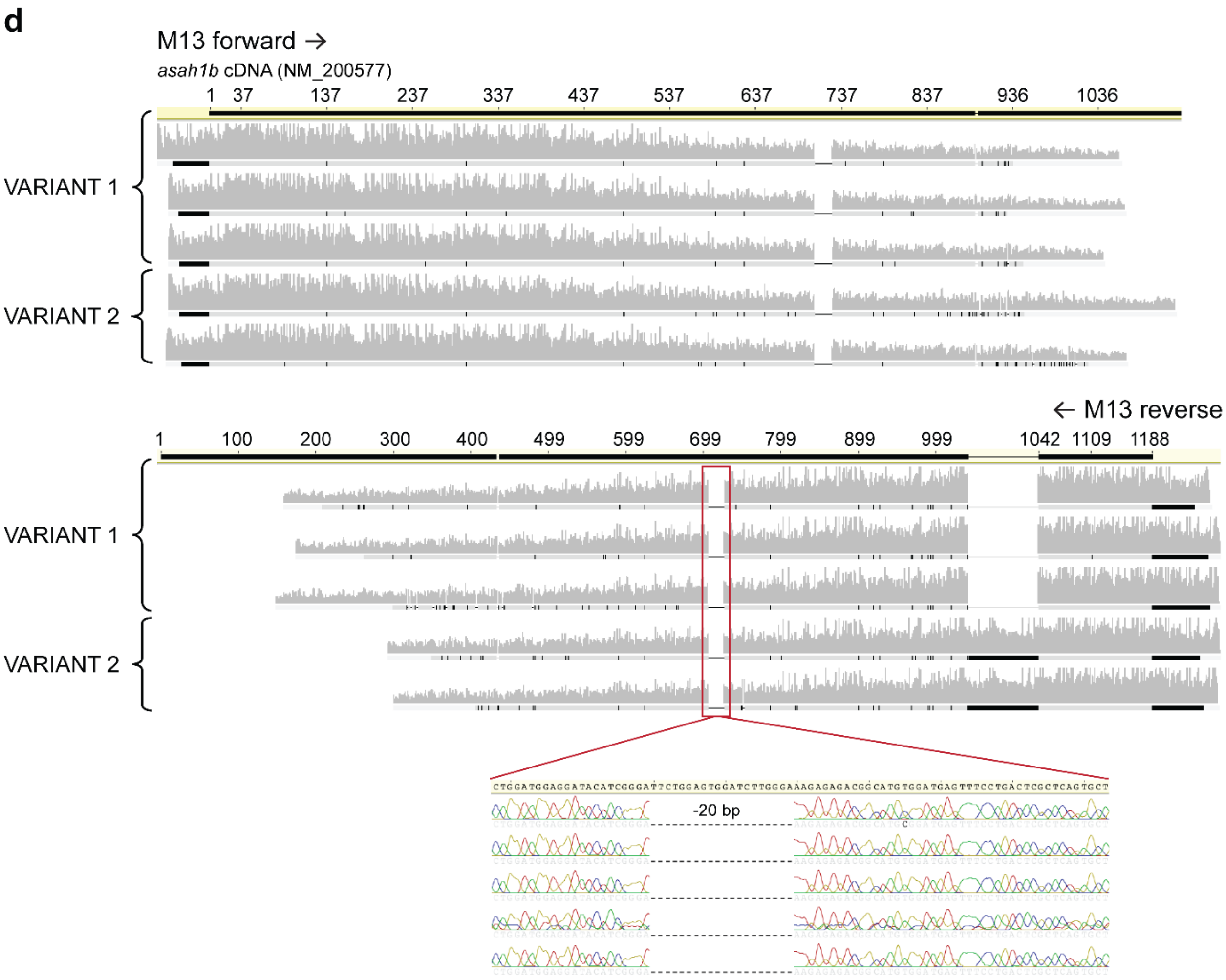

Figure S3. cDNA sequencing of Farber disease zebrafish. **(a)** Alignment of full-length *asah1a* cDNA sequences against reference *asah1a* cDNA (NM_001006088)[1]. Sequencing results were from ten individual bacterial colonies originating from four KO zebrafish [fish 1-2: *asah1a*(+68/+68), *asah1b*(-20/+); fish 3-4: asah1a(+68/+), asah1b (-20/-20)]. Five different splicing variants were identified at position 695 (exon 10 start) of reference sequence. Additional insertions (boxed region) in variant 4 and 5 correspond to a splicing variant that completely preserves intron 2-3. (**b**) The inserted sequences at position 695 were aligned against the *asah1a* genomic sequence (GRCz11)[3], which reveals the inserted regions to be a combination of partial intron 9-10, partial exon 6 and partial intron 6-7. The 73-bp genomic insertion (**Fig. S2a**) includes regions from exon 6 and intron 6-7, while the additional 47-bp

insertion aligns with the end of intron 9-10, suggesting that the latter was the result of a splicing error. (**c**) Graphical depiction of the five *asah1a* splicing variants; close proximity of the genomic mutation to the intron-exon junction (**Fig. S2a**) likely resulted in multiple splicing errors upon translation. **(d)** Alignment of full-length *asah1b* cDNA sequences against reference *asah1b* cDNA (NM_200577)[1]. Sequencing results were from five individual bacterial colonies originating from three KO zebrafish [fish 1: *asah1a*(+68/+68), *asah1b*(-20/+); fish 2-3: asah1a(+68/+), asah1b (-20/-20)]. Expanded view of boxed region demonstrates consistent presence of the 20-bp deletion that was first identified during genomic DNA sequencing (**Fig. S2b**). The additional insertions in variant 2 correspond to a splicing variant that preserves intron 12-13. Intronic sequences were not observed during sequencing of WT alleles, suggesting that the preservation of entire introns in some mutant alleles could be additional splicing errors triggered by mutations near intron-exon junctions. Alignments were performed with BLAST[2] and Geneious.

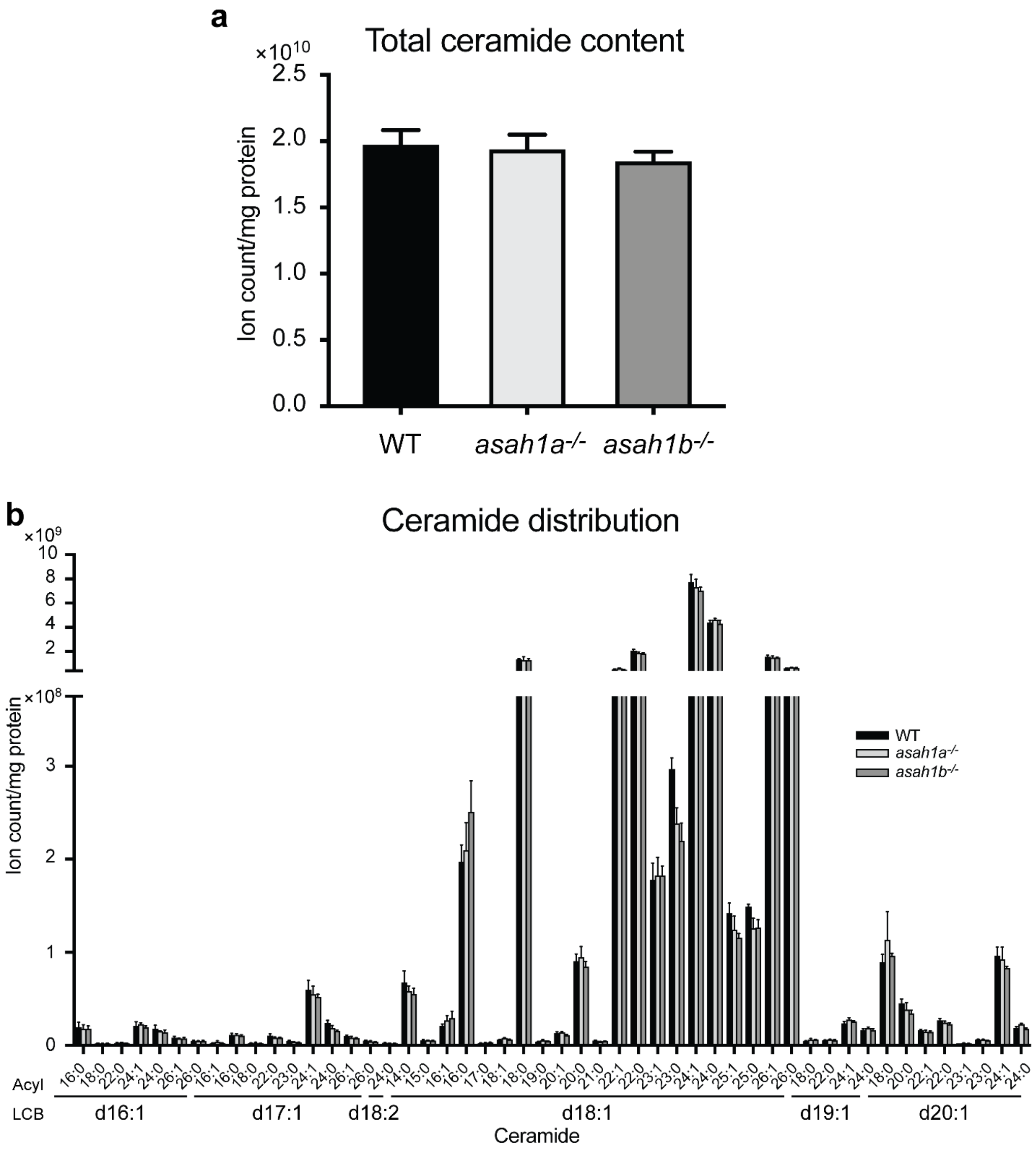

Figure S4. Ceramide content in wildtype and SKO zebrafish brains. (**a**) Total ceramide content in WT, *asah1a*$^{-/-}$ and *asah1b*$^{-/-}$ (SKO) zebrafish brains. (**b**) Individual ceramide levels across WT and SKO zebrafish brains. Ion count less than 10^5 were excluded due to low intensity. Student t-test, SEM, n=4 per group. Most ceramides were unchanged between WT and SKO populations.

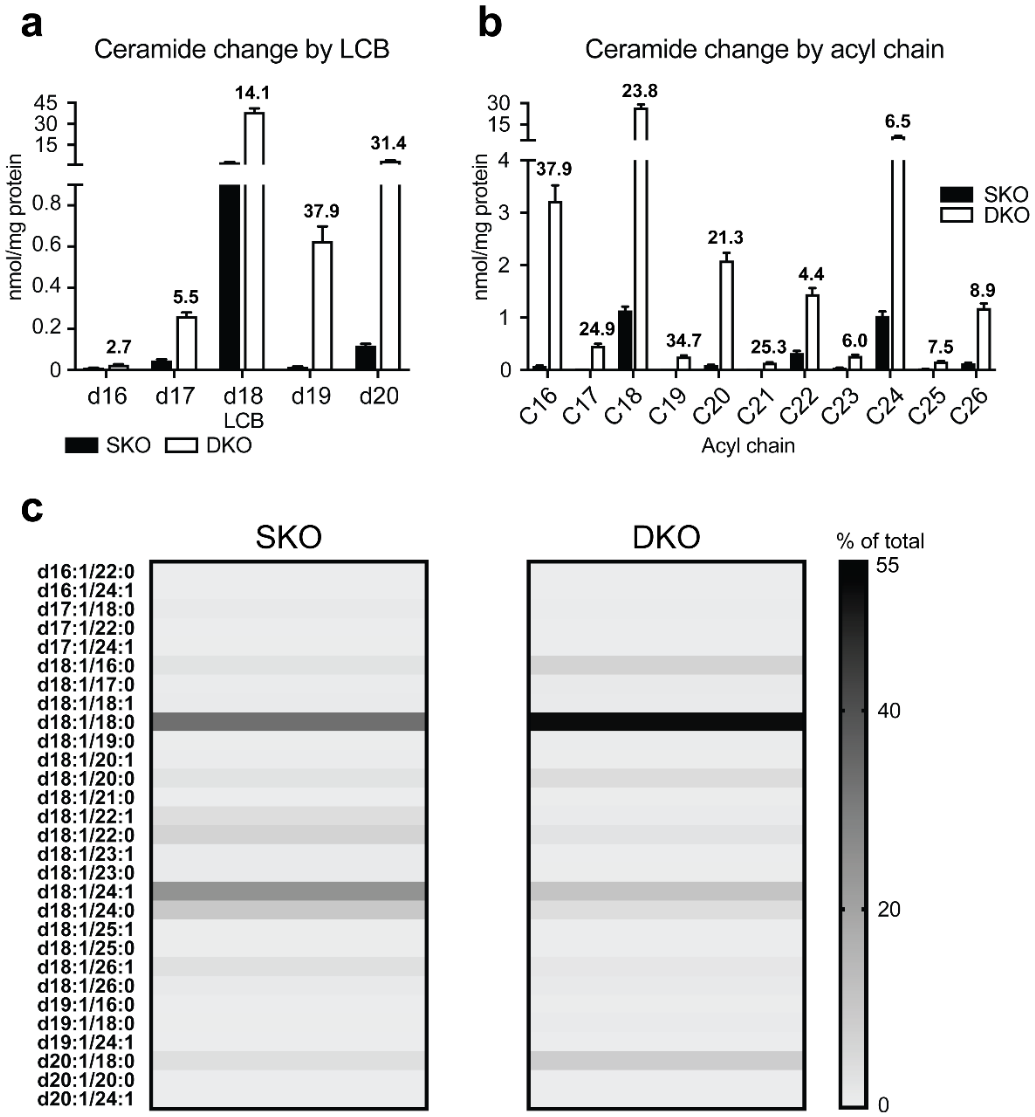

Figure S5. Altered ceramide distribution in Farber disease zebrafish. (**a**) Total ceramide content by LCB, DKO/SKO fold changes are indicated in graph. $p < 0.001$ for all LCBs. (**b**) Total ceramide content by acyl chain, DKO/SKO fold changes are indicated in graph. $p < 0.001$ for all acyl chains. (**c**) Percent distribution of all quantified SKO and DKO ceramide species. Only ceramides within linear range of the calibration curves (**Fig. S1**) are shown, see **Table S4** for all detected ceramides. Student t-test, SEM, n=4 per group. Representative data from two independent experiments are shown.

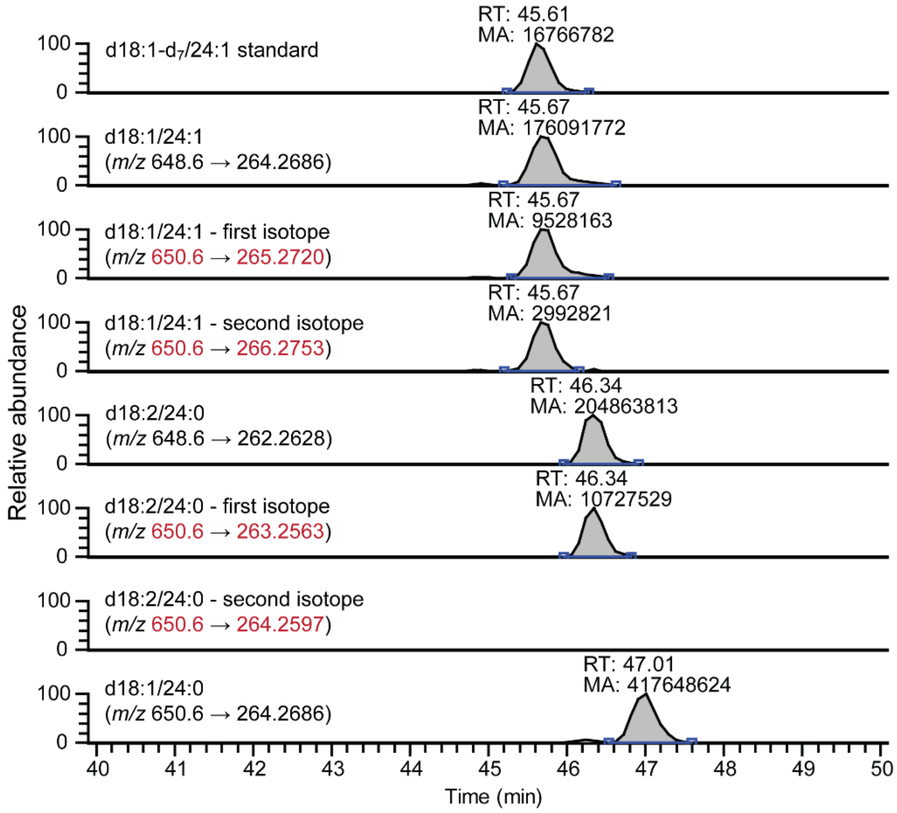

Figure S6. Additional notes on chromatography - part A. Extracted ion chromatograms of d18:2/24:0, d18:1/24:1 and d18:1/24:0 ceramides from HEK293 cells. Isotopic PRM transitions are in red. MA: peak area, RT: retention time.

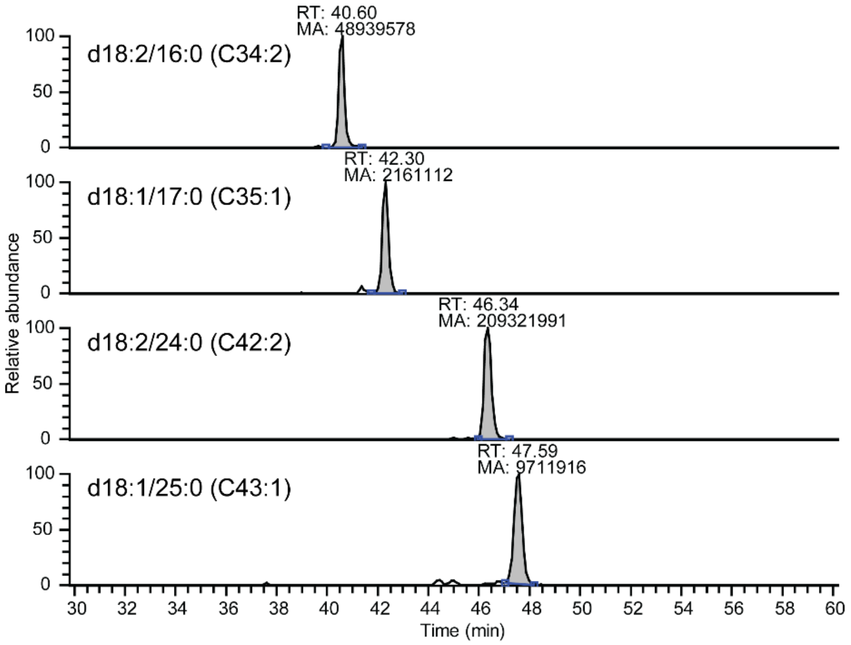

Figure S7. Additional notes on chromatography - part B. Extracted ion chromatograms of d18:2/16:0, d18:1/17:0, d18:2/24:0 and d18:1/25:0 ceramides from HEK293 cells, demonstrating resolution between each Cn:2 and C(n+1):1 ceramide pair. MA: peak area, RT: retention time.

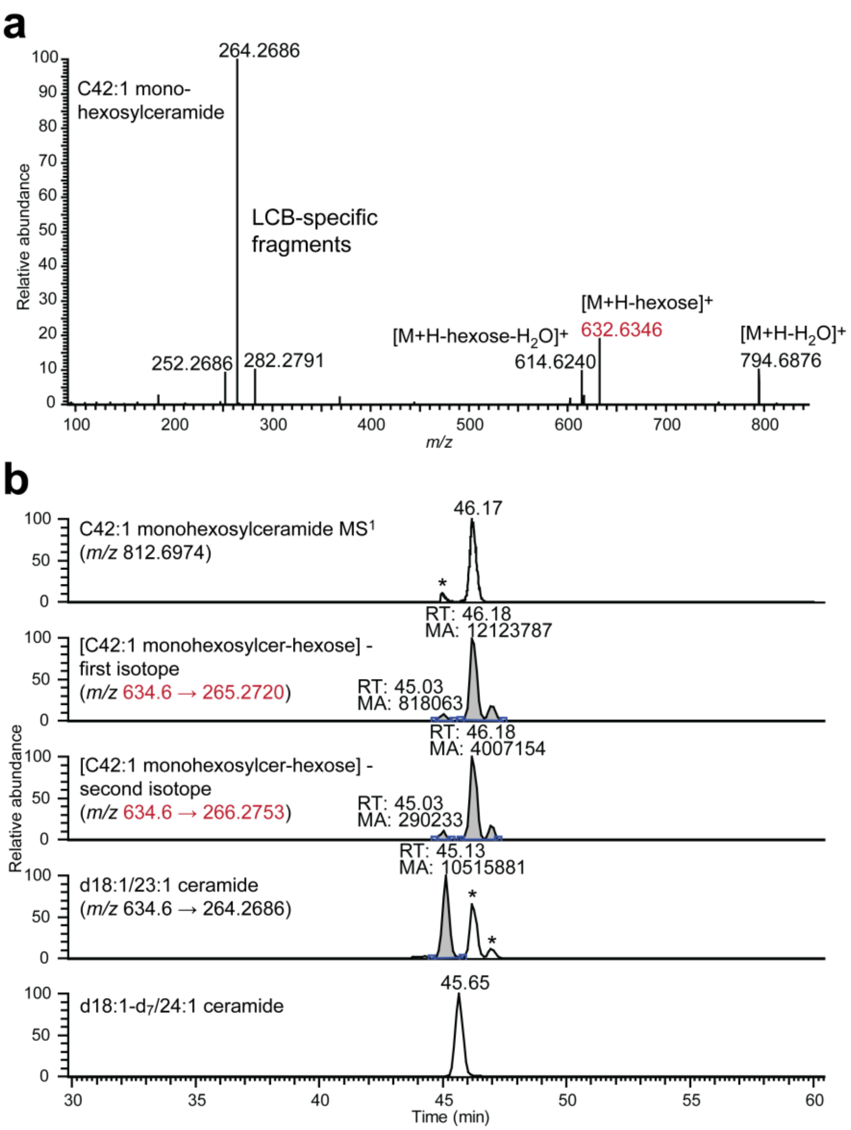

Figure S8. Additional notes on chromatography - part C. (**a**) MS^2 of C42:1 monohexosylceramide. The second isotope of the loss-of-hexose fragment (in red) overlaps with the parent ion of C43:2 ceramide in PRM mode. (**b**) Extracted ion chromatograms of C42:1 monohexosylceramide, isotopes of the loss-of-hexose adduct of C42:1 monohexosylceramide, C43:2 (d18:1/23:1) ceramide, and the d18:1-d_7/24:1 ceramide standard. Isotopic PRM transitions are in red. Asterisks denote contaminating isotopic peaks. Based on peak alignment and MS^2, the major nonspecific peak observed in the PRM spectrum of C43:1 ceramide is most likely derived from C43:1 monohexosylceramide. MA: peak area, RT: retention time.

a

LCB	Minor fragment 1	Major LCB fragment	Minor fragment 2	LCB	Minor fragment 1	Major LCB fragment	Minor fragment 2
d16:1	224.2373	236.2373	254.2478	d16:2	222.2216	234.2216	252.2322
d17:1	238.2529	250.2529	268.2635	d17:2	236.2373	248.2373	266.2478
d18:1	252.2686	264.2686	282.2791	d18:2	250.2529	262.2529	280.2635
d19:1	266.2842	278.2842	296.2948	d19:2	264.2686	276.2686	294.2791
d20:1	280.2999	292.2999	310.3104	d20:2	278.2842	290.2842	308.2948

b

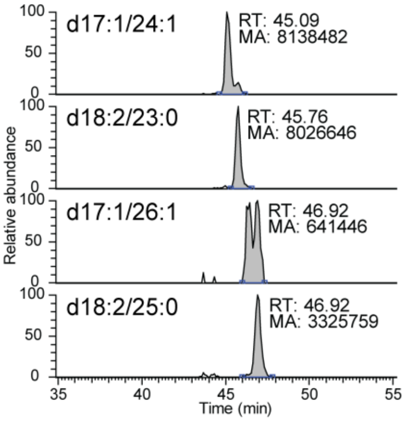

c

Ceramide	264/252	262/250	Major LCB fragments/ loss-of-water
C32:1	11.2		6.6
C33:1-d_7	11.3		5.8
C34:2		11.3	6.3
C34:1	11.1		6.7
C36:1	10.0		6.4
C38:1	11.4		6.1
C40:1	10.7		6.9
C42:2		11.5	6.7
C42:2-d_7	10.7		7.3
C42:1	10.6		7.9

Figure S9. Additional notes on chromatography - part D. (**a**) List of the major and two minor LCB-specific fragments for ceramide LCBs d16 through d20. Overlapping fragments from monounsaturated and diunsaturated LCBs are in red. (**b**) Extracted chromatograms of d17:1/24:1, d18:2/23:0, d17:1/26:1 and d18:2/25:0 ceramides. For each ceramide, some chromatographic separation is observed between each d17:1 and d18:2 isomer as to allow visual determination of the presence or absence of the dn:1 LCB; more accurate peak area calculation could be achieved *via* the background subtraction method described under Supplemental Discussion. (**c**) *m/z* 264/252, 262/250 and major LCB fragments/loss-of-water fragment ratios for selected HEK293 ceramides and ceramide standards; fragment ratios are independent of LCB/acyl chain lengths or degrees of unsaturation. For the third column, major LCB fragments are the sum of all detectable major LCB fragments for each ceramide species. MA: peak area, RT: retention time.

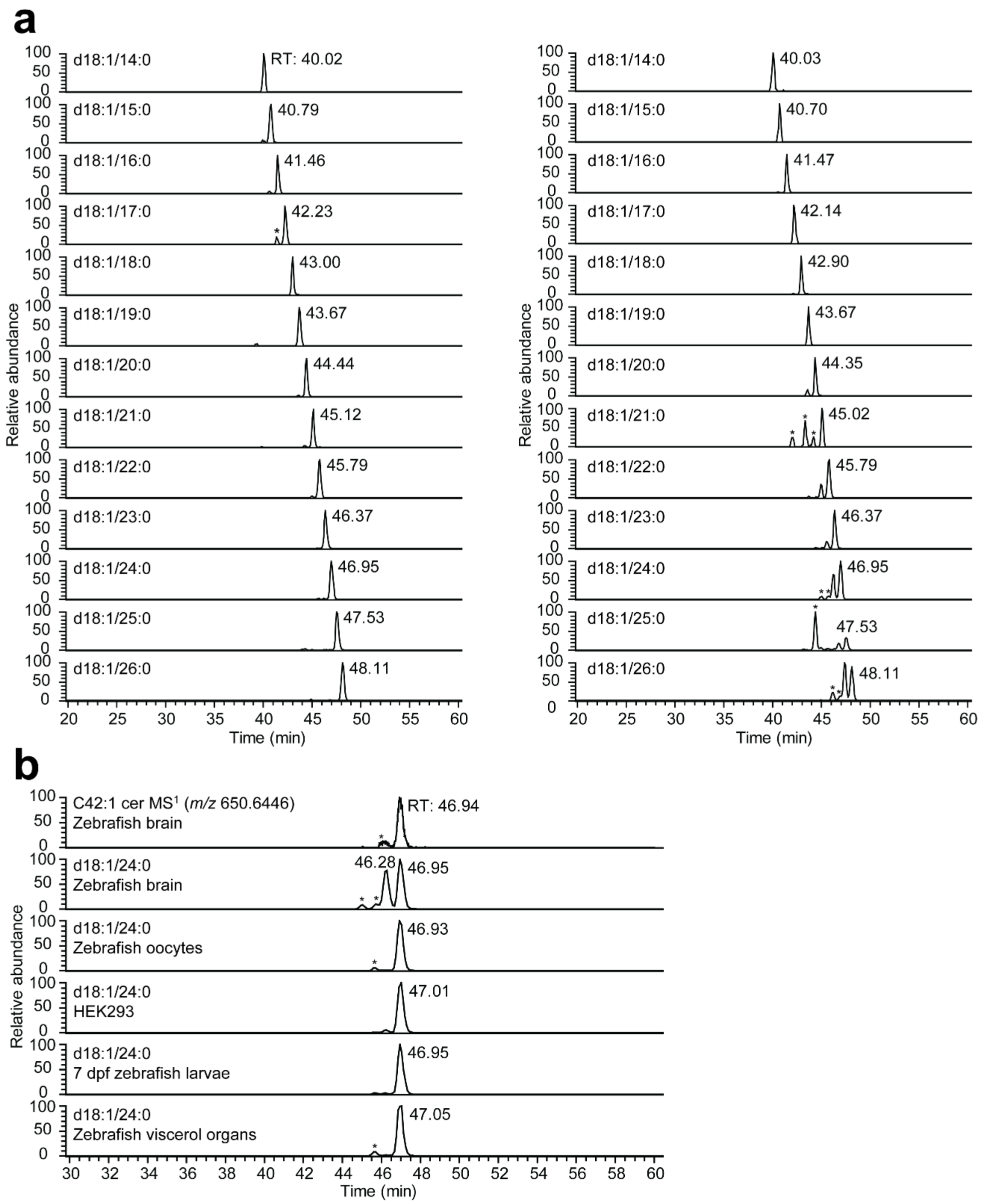
a
b
Relative abundance
Time (min)
d18:1/14:0
RT: 40.02
d18:1/15:0
40.79
d18:1/16:0
41.46
d18:1/17:0
42.23
d18:1/18:0
43.00
d18:1/19:0
43.67
d18:1/20:0
44.44
d18:1/21:0
45.12
d18:1/22:0
45.79
d18:1/23:0
46.37
d18:1/24:0
46.95
d18:1/25:0
47.53
d18:1/26:0
48.11
40.03
40.70
41.47
42.14
42.90
43.67
44.35
45.02
45.79
46.37
46.95
47.53
48.11
C42:1 cer MS1 (m/z 650.6446)
Zebrafish brain
RT: 46.94
d18:1/24:0
Zebrafish brain
46.28
46.95
d18:1/24:0
Zebrafish oocytes
46.93
d18:1/24:0
HEK293
47.01
d18:1/24:0
7 dpf zebrafish larvae
46.95
d18:1/24:0
Zebrafish viscerol organs
47.05

Figure S10. Additional notes on chromatography - part E. (**a**) All detectable d18:1 saturated ceramides from 7 dpf zebrafish larvae (left) and adult zebrafish brain (right). Note the appearance of doublet peaks in long-chain zebrafish brain ceramides. Asterisks denote nonspecific peaks. (**b**) Extracted ion chromatograms of d18:1/24:0 ceramide from adult zebrafish brain, oocytes from adult zebrafish, HEK293 cells, 7 dpf zebrafish larvae and adult zebrafish visceral organs. Asterisks denote nonspecific peaks. Note the presence of significant peak splitting exclusively in the brain PRM.

SUPPLEMENTAL DISCUSSION

Additional notes on chromatography

To ensure accurate quantification of ceramide isomers, potential isotope and fragment overlaps need to be considered. We discuss five chromatography-related issues associated with the current method as follows:

A. Overlap between monounsaturated ceramide and isotope of diunsaturated ceramide

In PRM mode, the lack of ppm mass accuracy at the MS^1 filter level leads to *m/z* overlap between the parent ion of each monounsaturated ceramide (i.e. C42:1) and the second isotope of the corresponding diunsaturated (i.e. C42:2) ceramide. In the event of identical retention time and equal signal strength between these two species, ~4-7 % of the monounsaturated ceramide MS^1 signal will contain the second isotopic ion from the diunsaturated ceramide (isotopic abundance predicted by ChemDraw).

In the context of our method, this issue is sufficiently minimised *via* baseline or close-to-baseline resolution between monounsaturated and diunsaturated ceramides. Taking the C42:2 species as example, in HEK293 cells this ceramide exists mostly as d18:2/24:0 and 18:1/24:1, both of which may overlap with the d18:1/24:0 PRM signal. For the d18:2/24:0 isomer, the overlapping ion in positive ionisation mode will be the second isotope of the major d18:2 LCB fragment originating from the second isotope of the d18:2/24:0 parent ion, and for the d18:1/24:1 isomer the overlapping ion will be the major d18:1 LCB fragment originating from the second isotope of the d18:1/24:1 parent ion (**Fig. S6**). Extracted chromatograms of C42:2 and C42:1 ceramides are in **Fig. S6**, demonstrating sufficient separation between d18:1/24:0 ceramide and the isotopes of d18:2/24:0 and d18:1/24:1 ceramides.

B. Overlap between diunsaturated ceramide and isotope of loss-of-water fragment of monounsaturated ceramide

In the absence of ppm mass accuracy, MS^1 overlap also occurs between any Cn:2 ceramide and the second isotope of the loss-of-water adduct of C(n+1):1 ceramide (i.e. C41:2 and C42:1-H_2O). Similar

to part A, this issue was minimalised *via* baseline or close-to-baseline resolution between all existing Cn:2 and C(n+1):1 pairs. Extracted chromatograms of C34:2 and C35:1 ceramides, and C42:2 and C43:1 ceramides, are in **Fig. S7**.

C. Overlap between diunsaturated ceramide and isotope of loss-of-water fragment of monohexosylceramide

As a follow up to part B, MS^1 overlap occurs between any Cn:2 ceramide and the second isotope of the loss-of-hexose adduct of C(n+1):1 monohexosylceramide (i.e. C41:2 ceramide and C42:1 monohexosylceramide-hexose). Positive mode MS^2 spectrum of C42:1 monohexosylceramide is in **Fig. S8a**, illustrating the presence of ceramide-specific fragments and a loss-of-hexose fragment at *m/z* 632.6346. In the absence of ppm mass accuracy, the second isotope of the hexosylceramide-derived *m/z* 632.6 fragment will also be detected in the C41:2 ceramide MS^1 channel under PRM mode. Similar to part B, this issue was minimised *via* baseline or close-to-baseline resolution between all Cn:2 ceramide and C(n+1):1 monohexosylceramide pairs, as is illustrated for C41:2 ceramide and C42:1 monohexosylceramide in **Fig. S8b**. Monohexosylceramide-associated peaks were present in the PRM spectra of C35:2, C41:2 and C43:2 ceramides in zebrafish larvae; C41:2 ceramide in HEK293 cells; and C41:2 and C43:2 ceramides in zebrafish brain. Appearance of monohexosylceramide peaks for the aforementioned ceramides is a likely reflection of the abundance of C36:1, C42:1 and C44:1 monohexosylceramides relative to other chain lengths.

While similar overlaps could also be expected for lactosylceramides and gangliosides, these species were rarely detected during untargeted analyses of our samples, as their increased hydrophilicity hinders extraction *via* the Bligh-Dyer-based method.

D. Major and minor LCB overlaps

A list of major and minor LCB-associated ceramide fragments is in **Fig. S9a**. *m/z*'s of overlapping fragments are in red. As illustrated, the major fragment of each dn:1 LCB shares the same

m/z as minor fragment 1 of the d(n+1):2 LCB, such that any ceramide composed of both dn:1 and d(n+1):2 LCBs will exhibit signal contamination in the dn:1 channel. For zebrafish, LCB overlap was avoided by the complete absence of ceramides containing quantifiable levels of both dn:1 and d(n+1):2 LCBs (**Table 1**), a result of the general lack of diunsaturated LCBs across zebrafish ceramides. Co-occurrence of dn:1 and d(n+1):2 LCBs was observed for two HEK293 ceramides (C41:2 ceramide: d17:1/24:1 and d18:2/23:0; C43:2 ceramide: d17:1/26:1 and d18:2/25:0) (**Table 1**, **Fig. S9b**).

To address this issue, we take advantage of the consistent ratio between MS^2 fragments across the ceramide profile (**Fig. S9c**), such that for any dn:1 LCB coexisting with the d(n+1):2 LCB, the amount of contaminating minor fragment signal could be estimated *via* the known ratio of major to minor fragments.

Taking d17:1/24:1 ceramide as example:

Adjusted area of d17:1/24:1 ceramide = area of d17:1/24:1 (*m/z* 634.6 → 250.2529) ceramide - [area of d18:2/23:0 (*m/z* 634.6 → 262.2529) ceramide]/{[major fragment of d18:1/23:0 (*m/z* 650.6 → 264.2686) ceramide]/[minor fragment 1 of d18:1/23:0 (*m/z* 650.6 → 252.2686) ceramide]}

In this case, the *m/z* 264/252 ratio from d18:1/23:0 ceramide was used to estimate the area of the *m/z* 250 fragment from d18:2/23:0 ceramide, given consistency of the *m/z* 264/252 ratio across ceramides with different acyl chain lengths and degrees of unsaturation (**Fig. S9c**).

E. Unidentified peaks in long chain monounsaturated zebrafish brain ceramides

During data analysis, we observed the appearance of doublet peaks for long chain monounsaturated zebrafish brain ceramides; prominent peak splitting was present for all monounsaturated brain ceramides from C22 onwards (**Fig. S10a**). Ceramide-specific MS^2 was present across both peaks. No doublet peaks were observed in HEK293 cells, zebrafish larvae or additional zebrafish tissues (**Fig. S10a, b**). This observation was surprising, as mature monounsaturated mammalian ceramides contain a

single *trans* double bond at the C4-C5 position of the LCB, thus minimizing the possibility of double bond isomers that may lead to peak splitting. Based on retention time alignment against additional sample types and the high-mass accuracy MS^1 spectrum (**Fig. S10b**), the first peak in each doublet was excluded from peak integration. Notably, only the second peak was elevated in the zebrafish Farber disease model, and inclusion of the first peak did not alter the data presented in **Fig. 5**.

While our current method did not allow identification of the additional peaks observed in zebrafish brain, MS^2 similarity across the two peaks suggests that the former could be a dihydroceramide with a diunsaturated LCB, or a hydroxylated ceramide that yields a ceramide fragment following loss-of-water in-source. It is worth noting that absence of majority of the first PRM peak from the high-mass accuracy MS^1 spectrum (**Fig. S10b**, top row) supports the identity of this peak as a ceramide derivative in which enough structural variation is present to significantly alter ionisation efficiency and/or in-source fragmentation behaviour. Importantly, the exact double bond locations of mature zebrafish ceramides remain unclear, though conservation of sphingolipid biosynthetic machinery across mammals and zebrafish supports structural similarities such as the 4,5-*trans* double bond. Additional fragmentation and separation methods could add further structural detail to the zebrafish sphingolipidome.

REFERENCES

1 O'Leary, N. A. *et al.* Reference sequence (RefSeq) database at NCBI: current status, taxonomic expansion, and functional annotation. *Nucleic Acids Res.* **44**, D733-D745; 10.1093/nar/gkv1189 (2016).

2 Altschul, S. F., Gish, W., Miller, W., Myers, E. W. & Lipman, D. J. Basic local alignment search tool. *J. Mol. Biol.* **215**, 403-410 (1990).

3 Zerbino, D. R. *et al.* Ensembl 2018. *Nucleic Acids Res.* **46**, D754-D761; 10.1093/nar/gkx1098 (2017).
